# Pharmacology-Driven Dissection of Core Component Sets of Xuefu Zhuyu Decoction in Blood Stasis-Related Cardiovascular Diseases

**DOI:** 10.3390/ph19040532

**Published:** 2026-03-25

**Authors:** Xuyang Dai, Dongsheng Ba, Miansheng Gao, Chen Liang, Ximeng Zhang, Huijuan Yu, Xin Chai, Yuefei Wang

**Affiliations:** 1 State Key Laboratory of Chinese Medicine Modernization, Tianjin University of Traditional Chinese Medicine, Tianjin 301617, China; 2 Haihe Laboratory of Modern Chinese Medicine, Tianjin 301617, China

**Keywords:** core active component set, cardiovascular diseases, blood stasis syndrome, Xuefu Zhuyu Decoction

## Abstract

Endothelial dysfunction, chronic inflammation, immune dysregulation, oxidative stress, mitochondrial dysfunction, and metabolic disturbances collectively contribute to cardiovascular diseases (CVDs) associated with blood stasis patterns. Xuefu Zhuyu Decoction (XFZYD) is widely used clinically for the management of CVDs. Based on serum-exposed prototype profiling in rats, two pharmacology-driven core component sets of XFZYD were defined as the core set for the promotion of blood circulation and the elimination of blood stasis (CPBEB; HSYA, GRo, FA, *β*-ECD, AMY, ALB, PF) and the core set for the regulation of qi and the relief of pain (CRQRP; LIQ, NR, NAR, ROF, HSD, NHP, LTG, NRG, ISL, FNT, NOB, PD, SSa). CPBEB primarily targets vascular pathology by regulating endothelial dysfunction with dyslipidemia-driven arterial lipid deposition. Mechanistically, CPBEB is associated with improved endothelial function, reduced plaque instability, attenuated chronic inflammation and oxidative stress, normalized lipid and bile acid metabolism, and decreased thrombosis. CRQRP primarily modulates vascular tone and systemic energy metabolism. These effects are linked to enhanced AMPK/SIRT1-driven antioxidant defenses and mitochondrial homeostasis, increased NO/cGMP signaling, coordinated crosstalk among the TLR4/NF-κB, JAK/STAT, NLRP3, and PPAR pathways, and remodeling of the gut microbiota–immune network. In summary, this review integrates modern analytical approaches with network pharmacology and the literature evidence to clarify the material basis underlying XFZYD’s therapeutic effects in CVDs, thereby supporting the modernization and internationalization of traditional Chinese medicine.

## 1. Introduction

Cardiovascular diseases (CVDs) remain a leading cause of mortality and long-term disability worldwide, imposing substantial clinical and socioeconomic burdens owing to high recurrence rates and chronic complications. Modern pharmacological studies indicate that blood stasis syndrome constitutes a fundamental pathological basis for a wide range of disorders, including CVDs, cerebrovascular diseases, gastrointestinal diseases, and metabolic disorders. Accordingly, promoting blood flow, improving microcirculation, and resolving blood stasis are key therapeutic principles for blood stasis syndrome.

Xuefu Zhuyu Decoction (XFZYD) is a classical traditional Chinese medicine prescription used to promote blood circulation, resolve blood stasis, regulate qi, and relieve pain. To meet diverse clinical needs, XFZYD has been developed in multiple dosage forms, including decoction, tablet, oral liquid, capsule, soft capsule, and pill. XFZYD primarily contains flavonoids, alkaloids, saponins, organic acids, terpenoids, and related constituents. Bibliometric analyses identify CVDs as the most prominent disease associated with XFZYD [1]. Consistently, accumulating clinical and preclinical evidence suggests that XFZYD may confer cardiovascular benefits by alleviating ischaemic symptoms and improving cardiometabolic risk profiles. Mechanistically, the existing research indicates that XFZYD exerts its therapeutic effects on CVDs through vascular and endothelial function, platelet activity, inflammation, oxidative stress, and glucose–lipid metabolism, which supports its importance in the treatment of CVDs [2,3,4,5].

Despite mounting evidence suggesting the cardiovascular benefits of XFZYD, a fundamental problem remains: its pharmacodynamic material basis and precise mechanism of action are not fully elucidated. Therefore, based on the high centrality of CVDs in bibliometric networks and existing clinical evidence [1], we define CVDs as the primary indication and construct the following research design and analytical framework: (i) integrating key CVDs’ pathological processes to construct a mechanistic framework; (ii) based on the mechanistic framework and combined with modern analytical methods, identifying and characterizing the core bioactive components of XFZYD. These core sets are proposed to underpin its traditional actions—the promotion of blood circulation and the elimination of blood stasis, and the regulation of qi and the relief of pain—and to link these actions to modern pharmacological mechanisms relevant to CVD therapy.

## 2. Literature Search Strategy

We conducted a structured literature search to identify studies on XFZYD in CVDs, with a particular focus on blood stasis-related contexts. The primary databases searched included CNKI, Wanfang, PubMed, and Web of Science. Google Scholar and SpringerLink were additionally used for supplementary searching and reference screening. Search terms combined XFZYD-related keywords with disease/mechanism terms: (“Xuefu Zhuyu” OR “Xuefu Zhuyu Tang” OR “Xuefu Zhuyu Decoction”) AND (“cardiovascular” OR “coronary” OR “atherosclerosis” OR “myocardial infarction” OR “angina” OR “hypertension” OR “ischemia”) AND (“blood stasis”).

All records were imported into a reference-management software for deduplication (automatic matching based on title, authors, year, journal, and DOI, followed by manual verification). Screening was then performed in two stages: first, titles and abstracts were assessed to exclude articles unrelated to XFZYD or CVDs; secondly, full texts of potentially eligible articles were reviewed to determine final inclusion according to prespecified inclusion and exclusion criteria. Any disagreements during screening were resolved by consensus between the two reviewers; if consensus could not be reached, a third reviewer was consulted for adjudication. Studies were included if the intervention was explicitly XFZYD (or a clearly specified standardized formulation/derivative dosage form of XFZYD) and if they reported cardiovascular-related evidence on efficacy, mechanisms, quality control, or safety. For clinical studies, a clear diagnosis based on recognized guidelines or diagnostic criteria was required. Studies were excluded if the composition of XFZYD or intervention details were unclear; if XFZYD was co-administered with multiple formulas such that its effects could not be reasonably attributed; if modified versions (addition/subtraction of herbs) could not be mapped to the original prescription; if the study was unrelated to CVDs; if only an abstract was available, the full text was unavailable, or key data were insufficient; or if the publication was duplicated.

## 3. Pathogenesis of Cardiovascular Diseases Associated with Blood Stasis Syndrome

The key messages of the Report on Cardiovascular Health and Diseases in China (2023) indicate that the burden of CVDs continues to increase, with an estimated 330 million people affected. Mortality rates in rural and urban areas are 48.00% and 45.86%, respectively. This trend imposes a substantial economic burden. However, CVD pathogenesis is highly complex and involves the interplay of multiple pathophysiological processes. Therefore, elucidating the mechanisms underlying CVDs is critical for prevention and treatment.

### 3.1. Endothelial Dysfunction and Vascular Remodeling

Endothelial dysfunction is a central early event in atherosclerosis and other CVDs. In the presence of major cardiovascular risk factors, reduced NO bioavailability together with increased ET-1 production shifts the vascular milieu toward vasoconstriction, heightened permeability, and a pro-inflammatory/pro-thrombotic state, thereby facilitating lipid entry and inflammatory mediator infiltration into the arterial wall [6,7]. Consistent with this activated endothelial phenotype, ox-LDL can induce upregulation of adhesion molecules (ICAM-1 and VCAM-1), promoting leukocyte recruitment and contributing to foam-cell formation and lesion development. Under inflammatory conditions, endothelial antigen-presenting capacity (e.g., MHC II expression) may further amplify local adaptive immune responses [8,9,10].

Vascular smooth muscle cells (VSMCs) contribute to vascular remodeling by undergoing phenotypic switching from a contractile to a synthetic state in response to inflammatory cytokines (e.g., TNF-α and IL-1β) and growth factors. This switch promotes VSMC proliferation and migration and increases extracellular matrix production (e.g., collagen and elastin), supporting fibrous cap formation. While the fibrous cap can partially stabilize plaques, persistent inflammatory signaling sustains endothelial–VSMC–immune crosstalk (including increased cytokines/chemokines such as IL-6, MCP-1 and adhesion molecules), thereby disrupting VSMC function, promoting chronic inflammation within lesions, and contributing to progressive vascular wall thickening and stiffening [11,12,13].

### 3.2. Chronic Inflammation and Immune Dysregulation

Chronic inflammation plays a pivotal role in CVD progression by sustaining immune-cell activation, promoting the persistent release of inflammatory mediators, and driving vascular structural remodeling.

Within lesions, macrophages play a central role in lipid handling and inflammatory amplification. Under pro-inflammatory cues (e.g., Th1-type cytokines and LPS), macrophages tend to polarize toward an M1-like phenotype, whereas Th2-associated environments favor M2-like programs that support inflammation resolution and tissue repair [14]. During atherosclerosis development, macrophages internalize ox-LDL via scavenger receptors (e.g., CD36 and SR-A), contributing to foam-cell formation. Excessive lipid uptake is associated with increased oxidative stress, further endothelial injury, and amplification of local inflammation; macrophage apoptosis/necrosis can contribute to necrotic-core formation and plaque burden [15,16].

Neutrophils can exacerbate oxidative injury and thrombo-inflammatory responses, including through ROS generation and NET-associated procoagulant effects, while also contributing to extracellular matrix degradation and plaque instability via proteases and oxidant-generating enzymes (e.g., MPO) [17]. Adaptive immune cells further shape inflammatory tone: pro-inflammatory T-cell cytokines (e.g., IFN-γ, TNF-α, and IL-17) activate macrophages and vascular cells and can aggravate lesion progression, whereas regulatory T cells (Tregs) secrete anti-inflammatory mediators (e.g., IL-10 and TGF-β) that restrain inflammation and may help maintain plaque stability [18,19]. In metabolic disorders such as insulin resistance/obesity, shifts in T-cell balance (reduced Tregs with increased Th1/Th17 activity) can intensify inflammation and vascular injury [20].

### 3.3. Oxidative Stress

Oxidative stress contributes to endothelial injury, VSMC dysfunction, and vascular remodeling in CVDs. Excessive ROS can compromise endothelial barrier integrity and increase vascular permeability, while activating pro-inflammatory signaling and elevating cytokine expression, thereby promoting inflammatory cell recruitment and plaque progression [21]. ROS also facilitate VSMC proliferation and migration through stress-responsive kinase pathways, reinforcing vascular remodeling [22].

Oxidative stress can also disturb calcium homeostasis (promoting Ca^2+^ overload) and increase risks of cardiomyocyte apoptosis and arrhythmogenesis [23]. In addition, ROS-driven activation of matrix-degrading enzymes can weaken fibrous cap stability and increase plaque rupture susceptibility. Autophagy impairment under oxidative stress may further hinder clearance of damaged mitochondria and misfolded proteins, exacerbating cellular injury [24].

### 3.4. Energy Metabolism Disorder

Insulin resistance (IR) promotes CVD progression through dysregulated lipid and glucose metabolism, oxidative stress, and inflammation. Reduced insulin-mediated antilipolysis, increases free fatty acid release, enhancing hepatic triglyceride synthesis and VLDL production and contributing to hyperlipidemia with increased LDL/ox-LDL, thereby sustaining lipid substrate availability for plaque formation [25]. Hyperglycemia and hyperlipidemia also promote advanced glycation end product (AGE) formation; AGE–RAGE signaling induces oxidative stress and inflammatory responses and contributes to vascular stiffness and accelerated atherosclerosis [26].

IR can reduce glucose utilization in cardiomyocytes and increase reliance on fatty acid oxidation, which elevates mitochondrial workload and ROS generation, promoting lipotoxicity, energy imbalance, cardiac hypertrophy, fibrosis, and heart failure [27,28]. IR also activates RAAS, contributing to elevated blood pressure and vascular remodeling. Furthermore, IR is closely associated with low-grade chronic inflammation: increased macrophage infiltration and a shift toward pro-inflammatory programs in adipose tissue enhance cytokine release (e.g., TNF-α and IL-1β), impair insulin signaling, and reinforce a vicious cycle linking metabolic disturbance, inflammation, and vascular injury [29,30].

### 3.5. Coagulation Function

Endothelial dysfunction diminishes the anticoagulant and anti-adhesive properties of the endothelium, exposing subendothelial pro-thrombotic components and triggering platelet adhesion, activation, and aggregation. Activated platelets release soluble mediators that amplify platelet recruitment and aggregation and can promote immune-cell adhesion and inflammatory activation, thereby aggravating endothelial injury and reinforcing thrombo-inflammatory loops [31,32].

Coagulation pathway activation results in thrombin generation and fibrin formation, stabilizing thrombi while further activating platelets and endothelial cells [33]. Following plaque rupture or erosion, abrupt exposure of tissue factor (TF) markedly accelerates coagulation and thrombosis, providing an immediate pathological basis for acute myocardial infarction and ischemic stroke.

### 3.6. Epigenetic Factors

Epigenetic regulation modulates cardiovascular-relevant gene expression programs and can influence endothelial function, vascular remodeling, and inflammatory activation during atherosclerosis. For example, DNA methylation changes (such as hypermethylation at the *NOS3* promoter) are associated with reduced eNOS expression and NO bioavailability, contributing to endothelial dysfunction [34]. Histone modifications shape chromatin accessibility and can bias inflammatory and vascular remodeling responses (e.g., through altered HDAC/HAT-related regulatory programs). Non-coding RNAs (including microRNAs and lncRNAs) further fine-tune endothelial and VSMC phenotypes, leukocyte adhesion, cholesterol handling, and angiogenesis-related processes [35,36]. Oxidative stress can interact with epigenetic regulation, potentially reinforcing inflammatory and endothelial dysfunction programs.

### 3.7. Gut Microbiota

The gut microbiota (GM) influences cardiovascular homeostasis by generating bioactive metabolites from dietary components and modulating immune and metabolic pathways. Short-chain fatty acids (SCFAs) are key microbial metabolites implicated in endothelial function, vascular tone regulation, and lipid metabolism, partly through GPCR signaling (e.g., GPR41 and GPR43) [37]. The gut microbiota also participates in amino acid metabolism, affecting downstream metabolites such as homocysteine, which is associated with increased atherosclerosis and CVD risk, whereas arginine-derived NO supports vasodilation and blood pressure regulation.

During dysbiosis, impaired intestinal barrier integrity can increase the translocation of gut-derived endotoxins, activate innate immune receptors (e.g., TLRs), and drive systemic low-grade inflammation and endothelial injury, thereby accelerating atherosclerosis. In addition, as microbial metabolite of nutrients, TMA is generated from choline/carnitine, which is converted to TMAO in the liver. Elevated TMAO is associated with plaque formation and CVD progression [38]. Gut microbiota–bile acid interactions influence signaling via receptors such as FXR and TGR5, linking microbial ecology to lipid metabolism, glucose homeostasis, and inflammatory responses.

## 4. XFZYD in the Treatment of Cardiovascular Diseases

### 4.1. Analysis of the Therapeutic Actions of the XFZYD Formula

In TCM, CVDs are often interpreted as a pattern of root deficiency and branch excess, in which deficiencies in qi, blood, yin, and yang predispose to manifestations such as qi stagnation, blood stasis, and collateral obstruction. XFZYD is a classical formula indicated for this pattern and is traditionally used to invigorate blood and regulate qi. From the perspective of contemporary medicine, although standard interventional therapies substantially improve outcomes, residual risk and treatment-related limitations remain. In this context, evidence from randomized controlled trials and meta-analyses suggest that adjunctive XFZYD added to standard therapy may improve CVD-related clinical and functional parameters and may favorably modulate inflammatory status and lipid profiles, with adverse reactions generally reported as mild in the available trials [3].

### 4.2. Analysis of the Core Component Sets of XFZYD

The core component sets were defined using the following criteria. (i) UPLC–Q–TOF/MS identified 24 prototype constituents in rat serum after 7 consecutive days of intragastric XFZYD administration [39]. (ii) The relevance of these 24 serum-exposed constituents to CVDs was assessed using DrugBank and TCMSP. Network pharmacology analyses were then performed to identify putative targets and pathways. Based on mechanistic features, the constituents were categorized into the core set for the promotion of blood circulation and the elimination of blood stasis (CPBEB) and the core set for the regulation of qi and the relief of pain (CRQRP). On this basis, 21 compounds were retained as the material basis of the CPBEB and CRQRP core sets, excluding *p*-hydroxycinnamic acid, *p*-hydroxybenzoic acid, and 18 *β*-glycyrrhizic acid. (iii) The therapeutic mechanisms of CPBEB and CRQRP against CVDs were reviewed and validated through literature searches in Web of Science and PubMed.

#### 4.2.1. Core Component Set for the Promotion of Blood Circulation and the Elimination of Blood Stasis

The CPBEB herb group—represented by *Persicae Semen* (Taoren), *Carthami Flos* (Honghua), *Chuanxiong Rhizoma* (Chuanxiong), *Angelicae Sinensis Radix* (Danggui), *Rehmanniae Radix* (Dihuang), *Paeoniae Radix Rubra* (Chishao), and *Achyranthis Bidentatae* (Niuxi)—primarily targets the core pathogenesis of the blood stasis pattern, namely impaired blood flow and obstruction of the channels and collaterals. By improving hemorheological properties, this group alleviates blood stasis-related manifestations (e.g., pain, a dark or dusky complexion, and palpable masses) and promotes collateral patency and tissue repair, thereby contributing to the overall therapeutic efficacy of XFZYD. The CPBEB core set comprises seven constituents: hydroxysafflor yellow A (HSYA), ginsenoside Ro (GRo), ferulic acid (FA), *β*-ecdysterone (*β*-ECD), amygdalin (AMY), albiflorin (ALB), and paeoniflorin (PF) (Table 1).

#### 4.2.2. Core Component Set for the Regulation of Qi and the Relief of Pain

The CRQRP herb group mainly comprises *Bupleuri Radix* (Chaihu), *Aurantii Fructus* (Zhiqiao), *Platycodonis Radix* (Jiegeng), and *Glycyrrhizae Radix et Rhizoma* (Gancao). It primarily targets upstream pathological links of the blood stasis pattern, namely stagnation of qi dynamics and dysregulation of qi–blood circulation. By regulating qi to facilitate blood flow, unblocking the collaterals, and relieving pain, this group acts synergistically with CPBEB herbs, accelerating the resolution of existing stasis and helping to prevent new stasis formation. This group reflects the TCM principle that “when qi moves, blood moves; when qi stagnates, blood stasis ensues” and, at a systemic level, may enhance the overall efficacy of XFZYD for blood stasis patterns and related cardio-cerebrovascular diseases. Relevant studies suggest that these herbs are enriched in triterpenoid saponins, flavonoids, and glycosides. These constituents are proposed to harmonize qi dynamics, soothe the liver and relieve constraint, and regulate the ascending and descending functions of the spleen and stomach, thereby promoting qi–blood flow and channel–collateral patency (Table 1).

#### 4.2.3. Network Pharmacology Analysis of the XFZYD Core Component Sets

Disease-related targets for CVDs were retrieved from public disease–target resources, including the Therapeutic Target Database (TTD), OMIM, and DisGeNET. Putative targets of XFZYD constituents were obtained from DrugBank, SwissTargetPrediction, and TCMSP. Targets from all sources were merged, deduplicated, and standardized to official gene symbols before downstream network construction and GO/KEGG enrichment analyses.

**Figure 1:**
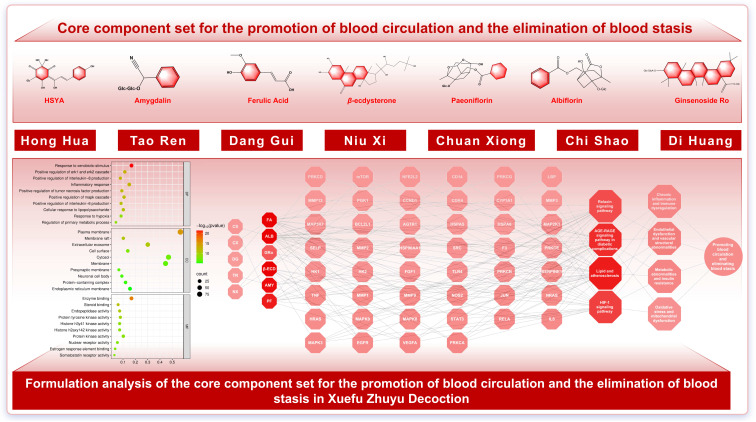
Network pharmacology analysis of the core component set for the promotion of blood circulation and the elimination of blood stasis. FA, Ferulic acid; ALB, Albiflorin; GRo, Ginsenoside Ro; *β*-ECD, *β*-ecdysterone; AMY, Amygdalin; PF, Paeoniflorin; TR, *Persicae semen*; CX, *Chuanxiong rhizoma*; CS, *Paeoniae radix rubra*; DG, *Angelicae sinensis radix*; NX, *Achyranthis bidentatae*.

**Figure 2:**
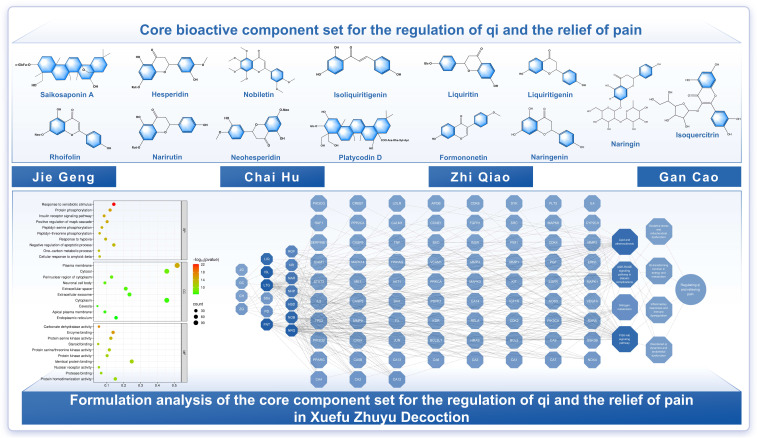
Network pharmacology analysis of the core bioactive component set for the regulation of qi and the relief of pain. JG, *Platycodonis Radix*; GC, *Glycyrrhizae Radix et Rhizoma*; CH, *Bupleuri Radix*; ZQ, *Aurantii Fructus*; LIQ, Liquiritin; ISL, Isoliquiritigenin; LTG, Liquiritigenin; SSa, Saikosaponin A; PD, Platycodin D; FNT, Formononetin; ROF, Rhoifolin; NR, Narirutin; NAR, Naringin; NHP, Neohesperidin; HSD, Hesperidin; NOB, Nobiletin; NRG, Naringenin.

GO enrichment analysis suggests that CPBEB may act by modulating membrane receptor–kinase signaling and nuclear receptor–transcriptional axes, thereby shaping inflammatory and stress responses, integrating stress and metabolic cues, and supporting cell survival. In addition, pathway enrichment analysis indicates that CPBEB may exert therapeutic effects on CVDs via pathways including lipid and AS, the AGE–RAGE signaling pathway in diabetic complications, relaxin signaling, and HIF-1 signaling. Collectively, these pathways may modulate endothelial dysfunction and vascular structural abnormalities, chronic inflammation and immune dysregulation, oxidative stress and mitochondrial dysfunction, and metabolic disturbances and insulin resistance (Figure 1). CRQRP may act predominantly via lipid and AS, the AGE–RAGE signaling pathway in diabetic complications, PI3K–Akt signaling, and nitrogen metabolism. Through these pathways, CRQRP may help regulate disordered qi dynamics and endothelial dysfunction, inflammatory responses and immune dysregulation, energy metabolism-related qi-transforming function, and oxidative stress and mitochondrial dysfunction, thereby contributing to CVD management (Figure 2).

### 4.3. Mechanisms of the Core Component Set for the Promotion of Blood Circulation and the Elimination of Blood Stasis

#### 4.3.1. Regulation of Endothelial Dysfunction and Vascular Structural Abnormalities

CPBEB acts on key processes, including endothelial function, lipid deposition, vascular tone, and barrier integrity. HSYA reduces ox-LDL uptake and deposition, thereby inhibiting the transformation of macrophages into foam cells. In addition, HSYA modulates eNOS expression and increases NO production, attenuating ox-LDL-induced injury in human coronary artery endothelial cells (HCAECs) and promoting endothelial repair. Ox-LDL stimulation activates the Piezo1 ion channel and triggers inflammatory responses; HSYA may attenuate AS progression by inhibiting Piezo1/JNK signaling [40]. Moreover, sphingosine kinases 1/2 (SphK1/2) catalyze sphingosine phosphorylation to generate sphingosine-1-phosphate (S1P). Through S1P receptors 1/3 (S1PR1/3), S1P modulates eNOS activity and vascular tone. HSYA improves endothelial barrier function and atherosclerotic lesions in ApoE^−/−^ mice by suppressing SphK1 expression, reducing S1P production, and inhibiting S1PR3 [41]. In the cerebrovascular system, HSYA interacts with molecules such as HIF-1α, BNIP3, and Notch1, thereby inhibiting excessive post-ischemic autophagy [42]. Under hypoxic conditions, HSYA blocks the HIF-1α/NOX2 cascade and preserves tight junction protein ZO-1 expression, thereby protecting blood–brain barrier integrity [43]. The combination of HSYA and quercetin may modulate blood–brain barrier integrity [44].

In rats fed a high-fat, high-cholesterol diet, FA reduces cardiovascular injury markers and vascular superoxide production. FA also suppresses the expression of TNF-α, ACE, AT1R, gp91^phox^, and VCAM-1, and attenuates excessive activation of MMP-2 and MMP-9, thereby limiting hypertrophic aortic wall remodeling and inflammatory cell adhesion [45]. Functionally, FA inhibits L-type Ca^2+^ channels and may reduce myosin light chain phosphorylation, thereby alleviating coronary vasospasm [46]. Furthermore, legumain-targeted FA–peptide nanofibers have been developed to enhance FA delivery and coordinately regulate macrophage–endothelial cell interactions. In MI models, this strategy yields combined anti-inflammatory, pro-angiogenic, and cardioprotective effects and promotes the recovery of cardiac function [47].

In HUVECs exposed to environmental risk factor-related injury, AMY reduces ROS levels, increases SOD and GSH, and lowers MDA. AMY also suppresses the release of inflammatory mediators, including IL-6, IL-1β, TNF-α, and COX-2. Mechanistically, AMY inhibits NF-κB p65 nuclear translocation and IκB-α phosphorylation and modulates the Bcl-2/Bax ratio, thereby attenuating endothelial cell injury [48]. In addition, AMY inhibits phosphorylation of MAPK1, MAPK8, and MAPK14 and suppresses AP-1 signaling, thereby potentially slowing AS progression [49].

ALB and PF are particularly involved in regulating endothelial barrier homeostasis, endothelial–mesenchymal transition (EndMT), and vascular remodeling. ALB has a high binding affinity for EGFR, inhibits EGFR/Akt phosphorylation, and attenuates apoptosis, oxidative stress, and mitochondrial damage under high-glucose/high-lipid conditions [50]. PARP1 is a DNA damage-sensing and repair enzyme that regulates multiple cellular programs and pathogenic processes, including diabetes-related cardiovascular complications. ALB targets PARP1 to inhibit NF-κB activation, and PARP1 overexpression reverses ALB-mediated protection in HUVECs [51]. In peripheral arterial disease, PF upregulates VEGFA, MMP2, MMP9, and ERα and activates ERα/ROCK2 signaling. PF promotes endothelial cell migration and tube formation and induces a shift in macrophage phenotype from pro-inflammatory to reparative, thereby alleviating excessive inflammation and supporting vascular remodeling and functional recovery [52]. RXRα, a transcription factor for VE-cadherin, is critical for maintaining endothelial homeostasis and conferring anti-inflammatory and anti-apoptotic effects. When endothelial injury downregulates VE-cadherin and disrupts barrier integrity, PF restores VE-cadherin expression via activation of the RXRα axis, thereby strengthening the endothelial barrier and conferring protection against AS and myocardial ischemia–reperfusion (I/R) injury [53].

In the context of vascular remodeling and EndMT, PF blocks monocrotaline-induced EndMT, restores endothelial marker expression, upregulates bone morphogenetic protein receptor 2 (BMPR2), and inhibits phosphorylation of TGF-β-activated kinase 1 (TAK1), thereby preventing activation of downstream MAPK/NF-κB signaling. In vitro, PF inhibits platelet-derived growth factor-BB (PDGF-BB)-induced proliferation and the migration of human pulmonary arterial smooth muscle cells (PASMCs) and reverses the transition of endothelial cells toward a mesenchymal-like phenotype induced by combined stimulation with TGF-β1, IL-1β, and TNF-α [54]. In a flap ischemia–necrosis model, PF activates Nrf2/heme oxygenase-1 (HO-1) signaling, suppresses excessive mitochondrial ROS generation, and stabilizes mitochondrial membrane potential, thereby protecting endothelial cells from oxidative injury [55].

CPBEB centers on key nodes, including ox-LDL handling, eNOS/NO signaling, the S1P–S1PR axis, HIF-1α/NOX2, EGFR/PARP1, RXRα/VE-cadherin, and EndMT–smooth muscle remodeling. Collectively, these nodes form an integrated regulatory network spanning lipid deposition, endothelial barrier function, and vascular remodeling. This network provides a multi-target, cross-pathway pharmacological basis for correcting endothelial dysfunction and vascular structural abnormalities in CVDs.

#### 4.3.2. Balance of Chronic Inflammatory Response and Immune State

Along the canonical NF-κB/MAPK inflammatory axis, HSYA downregulates iNOS, COX-2, and NF-κB, inhibits p38 and JNK phosphorylation, and concomitantly increases ERK phosphorylation [56]. FA reduces IKKβ and NF-κB phosphorylation, thereby suppressing downstream inflammatory gene transcription. In I/R injury, FA activates MEK/ERK signaling and inhibits cyclophilin D (CypD)-mediated opening of the mitochondrial permeability transition pore (mPTP), thereby decreasing ROS production and cytochrome c release and ultimately limiting apoptosis in brain microvascular endothelial cells [57,58]. GRo downregulates p38 and JNK phosphorylation within the MAPK pathway and concomitantly suppresses Bax/Caspase-3-mediated apoptosis and microglia- and astrocyte-driven neuroinflammation, thereby attenuating Aβ deposition and cognitive impairment in APP/PS1 mice [59]. ALB and AMY block NF-κB p65 phosphorylation and nuclear translocation and inhibit excessive activation of p38 MAPK/NF-κB, ERK, JNK, and TLR4/NF-κB signaling, thereby restraining inflammatory cascades across multiple disease models [60,61,62,63].

Along the JAK2/STAT3 cytokine signaling axis, HSYA activates JAK2/STAT3 and induces feedback upregulation of SOCS3, thereby limiting excessive cytokine release and attenuating inflammation-associated damage in focal cerebral ischemia [64]. By contrast, AMY modulates JAK2/STAT3 signaling, downregulates multiple inflammatory cytokines, and reshapes T-cell subset composition, suggesting a negative regulatory role in adaptive immune responses [65]. In parallel, a pH-responsive lipid–polymer hybrid nanoparticle delivery system for PF enables passive, inflammation-targeted release and modulates STAT signaling-mediated macrophage M1/M2 polarization, thereby yielding combined anti-inflammatory and tissue repair effects [66]. Rutin and PF upregulate STAT3 signaling in hippocampal and cortical neurons, helping to maintain synaptic homeostasis and an anti-inflammatory balance in the neuronal microenvironment [67].

Along the TLR4–NLRP3 inflammasome–pyroptosis axis, multiple core constituents may cooperatively suppress excessive innate immune activation. HSYA inhibits angiotensin II (Ang II)-induced intracellular TLR4 expression and NF-κB phosphorylation, prevents NF-κB nuclear translocation, and thereby suppresses NLRP3 inflammasome assembly. HSYA further attenuates cerebral I/R injury by inhibiting pyroptosis mediated by the NLRP3, AIM2, and Pyrin inflammasomes [68]. HSYA also inhibits PI3K and modulates AKT/mTOR and NF-κB signaling in macrophages, thereby regulating inflammation and inflammation-associated lymphangiogenesis and conferring protection against AS [69]. Large liposomal nanoparticles encapsulating HSYA and hybridized with macrophage membranes inhibit *Atg13* DNA methylation, promote macrophage autophagy, and accelerate cholesterol efflux. By enhancing HSYA targeting, these nanoparticles reduce macrophage infiltration into plaques and decrease MMP-9 expression. A plaque/macrophage dual-targeting strategy for HSYA promotes macrophage polarization from an M1 to an M2 phenotype, reduces plaque area, and increases plaque stability in AS [70]. Furthermore, competitive binding to the Lys-166 site of ZBP1 inhibits ZBP1–NLRP3 signaling and reduces macrophage recruitment [71]. Inhibition of the NLRP3/Caspase-1/GSDMD cascade may alleviate the pyroptosis-associated injury [72]. ALB suppresses NLRP3 expression and mitigates oxidative stress-induced inflammatory responses [73]. In models of metabolic dysfunction-associated fatty liver disease (MAFLD), PF inhibits the NLRP3 inflammasome and STING-mediated hepatocyte pyroptosis, thereby reducing hepatic lipid deposition and inflammatory cell infiltration [74].

In terms ofimmune-cell polarization and inflammatory microenvironment remodeling, the core constituents reprogram macrophages, microglia, and T-cell subsets, thereby helping to correct the immune imbalance associated with chronic inflammation. The FA-1a-CS/SF composite hydrogel improves FA biocompatibility, induces macrophage polarization from an M1 to an M2 phenotype, and reduces ROS production [75]. In hypoxia models, PF inhibits NF-κB/HIF-1α signaling, suppresses M1 polarization, and promotes M2 polarization, thereby helping to restore immune balance and attenuate fibrosis. PF has also been reported to modulate gasdermin D (GSDMD) activity and reduce PI3K and Akt phosphorylation, thereby attenuating Ang II-induced atrial fibrosis and atrial fibrillation [76,77]. ALB upregulates NURR1, a negative regulator of inflammatory transcription, and inhibits P2X7 receptor signaling, thereby reducing epithelial–mesenchymal transition (EMT) in hepatic stellate cells, limiting extracellular matrix (ECM) deposition, and lowering inflammatory cytokine levels [78].

In the context of neuroimmune–metabolic crosstalk, metabolomic studies suggest that ALB and PF ameliorate multiple metabolic perturbations in the cerebral cortex and serum, including ether lipids, amino acids (alanine, aspartate, glutamate, tryptophan, arginine, and proline), carnitines, and arachidonic acid. ALB and PF also modulate hypothalamic–pituitary–adrenal axis function, thereby improving central immune–metabolic homeostasis [63]. PF inhibits TLR4/MYD88/NF-κB signaling and the NLRP3 inflammasome, attenuates inflammatory responses and microglia-mediated injury, protects midbrain dopaminergic neurons, and helps maintain neuronal microenvironment stability [79]. PF also ameliorates cognitive impairment by modulating AGEs–RAGE signaling and MAPK/mTOR-related autophagy defects [80]. PF directly binds *MAPK8* (JNK1) and *MAPK9* (JNK2), inhibits JNK activation, and reduces apoptosis [81].

CPBEB acts through key inflammatory–immune networks, including NF-κB/MAPK, JAK2/STAT3, the TLR4/NLRP3 inflammasome, and immune-cell polarization. Collectively, these networks constitute an integrated regulatory framework for chronic inflammation and immune dysregulation across cardiovascular, neurological, and metabolic diseases.

#### 4.3.3. Antioxidant Defense and Mitochondrial Homeostasis

HSYA activates the HIF-1α/SLC7A11/GPX4 axis to inhibit ferritinophagy and ferroptosis, thereby restoring redox homeostasis, reducing I/R-induced neuronal death, and suggesting potentially benefiting cardio-cerebrovascular diseases [82,83]. Complexation of FA and quercetin with *β*-glucan increases stability and functional activity. In DPPH, ABTS, and hydroxyl radical scavenging assays, these complexes show enhanced antioxidant capacity and reduced free radical generation [84,85]. FA activates Nrf2/HO-1 signaling while inhibiting NF-κB and TGF-β/Smad3 pathways, downregulates AChE and BACE, reduces GFAP expression, and corrects cholinergic and glutamate/GABA imbalance, thereby exerting neuroprotective effects across multiple neural injury models [47,86,87,88,89,90]. In combination with PF, *β*-ECD modulates Nrf2/HO-1 and SLC7A11/GPX4 signaling and reverses hypertrophy-induced abnormalities in cell surface area, ANP and β-MHC expression, MDA and SOD levels, calcium homeostasis, and mitochondrial membrane potential [91]. *β*-ECD may act in part by activating Akt signaling and promoting nuclear accumulation of Nrf2, thereby contributing to HO-1 induction and suppression of ROS production [92,93]. By preventing activation of the ROS-dependent ASK1–p38 MAPK pathway, *β*-ECD protects mitochondria and modulates p53 expression [94]. In addition, *β*-ECD regulates Bcl-2/Bax expression and thereby influences neuronal apoptosis [95]. AMY upregulates Nrf2, catalase, SOD-2, and GPX-4. By contrast, ALB binds Keap1 and regulates AKT/Nrf2/HO-1 signaling and PGK1 degradation, thereby strengthening endogenous antioxidant defenses and exhibiting cardio- and neuroprotective potential in models of exercise-induced myocardial ischemia/hypoxia and cerebral ischemia [96,97,98,99,100]. Collectively, these findings suggest that ALB may be a promising candidate for ischemic stroke; however, further pharmacokinetic and preclinical evaluation is required.

Dietary supplementation with FA promotes microglial polarization toward an M2 phenotype and suppresses inflammation. FA also activates the full-length 37-kDa form of APE1, promotes the repair of oxidative DNA damage, preserves mitochondrial function, and inhibits nuclear translocation of AIF, thereby reducing neuronal apoptosis [101].

HSYA has been reported to directly target the splicing factor SF3A1 and to covalently bind its Cys137 residue, thereby activating malate dehydrogenase 1 (MDH1) and restoring mitochondrial energy metabolism homeostasis. HSYA also improves mitochondrial function and energy supply in the ischemic penumbra via the PGC-1α/nuclear respiratory factor pathway [102,103,104]. Within the SIRT1/FOXO/PGC-1α signaling network, HSYA upregulates SIRT1, FOXO, PGC-1α, and Bcl-2 while suppressing Bax. Pharmacological inhibition or genetic deletion of *SIRT1* partially attenuates these anti-inflammatory and cytoprotective effects, indicating that SIRT1 is a regulatory hub through which HSYA maintains mitochondrial homeostasis and counters oxidative stress [105,106]. PF activates the SIRT1–PINK1/Parkin mitophagy axis and promotes selective clearance of damaged mitochondria, thereby alleviating myocardial I/R injury [107]. GRo enhances LKB1/AMPK phosphorylation and promotes nuclear colocalization of SIRT1 and PGC-1α, thereby augmenting autophagy/mitophagy and ATP production while reducing Aβ-induced ROS overproduction [108]. In metabolic syndrome-related phenotypes, FA improves disordered glucose and lipid metabolism via SIRT1/AMPK and AMPK/SREBP1/ACC1 signaling. FA also ameliorates mitochondrial dysfunction by promoting mitochondrial biogenesis, optimizing the fusion–fission balance, and restoring autophagy [109,110]. Overall, these constituents may converge on a splicing regulation–SIRT1/AMPK–PGC-1α–PINK1/Parkin axis, forming a multilayered protective network that links transcriptional and splicing regulation with mitochondrial dynamics and autophagy.

In addition, FA decreases ROS accumulation, inhibits NF-κB-mediated inflammatory responses, and modulates apoptosis-related factors, including Bax/Bcl-2, cytochrome c, caspase-3/9, and p53, thereby potentially influencing disease progression [111]. In models of ischemic brain injury, PF inhibits caspase-3/9 activation, maintains cytoplasmic localization of HDAC4, prevents cytochrome c release, and restores mitochondrial membrane potential, thereby conferring neuroprotective effects [112].

PF may serve as a key integrator of mitochondria-related programmed cell death pathways, including apoptosis, ferroptosis, and pyroptosis. It also activates Keap1/Nrf2/HO-1 and HSF1/NRF1 signaling, enhances antioxidant enzyme activity (SOD, GSH-Px, and CAT), reduces COX-2, iNOS, cleaved caspase-3/8, and pro-inflammatory cytokines, and upregulates IL-10, thereby conferring combined antioxidative, anti-inflammatory, and anti-apoptotic effects [113,114]. Along the ferroptosis axis, PF promotes p53 ubiquitination and proteasomal degradation and inhibits p53 acetylation, thereby weakening p53-mediated transcriptional repression of SLC7A11, preserving the SLC7A11–GPX4 antioxidant axis, and suppressing ferroptotic cell death [115]. In addition, PF modulates PI3K/AKT and MAPK signaling, thereby inhibiting ferroptosis and oxidative stress responses and protecting hepatocytes against estrogen-induced metabolic injury [116]. PF also inhibits GSDMD-dependent pyroptotic pathways and NLRP3 inflammasome activation, thereby reducing inflammatory cytokine release [117,118]. Together with its capacity to activate AMPK and upregulate ESR1, PF may establish an integrated regulatory network linking mitochondrial function, oxidative stress, and programmed cell death across multiple tissues, including the heart, brain, and liver.

CPBEB is organized around a continuous antioxidant–mitochondrial–ferroptosis axis and shows a consistent protective pattern across multiple cardio-cerebrovascular and metabolic disease models. This pattern highlights the formula’s multi-component, multi-target capacity to modulate oxidative stress and mitochondrial dysfunction across disease spectra.

#### 4.3.4. Improvement of Metabolic Abnormalities and Insulin Resistance

CPBEB coordinately regulates cholesterol and lipid metabolism, glucose homeostasis, and lipotoxicity. In obesity and type 2 diabetes (T2DM)-related contexts, safflower yellow and its major constituent, HSYA, inhibit the excessive secretion of glucose-dependent insulinotropic polypeptide (GIP), thereby improving body weight and metabolic disturbances in diet-induced obese mice [119]. GRo targets the bile acid receptor TGR5, promotes GLP-1 secretion, and enhances energy expenditure, thereby attenuating high-fat diet (HFD)-induced obesity and metabolic syndrome-like phenotypes [120]. In addition, HSYA contributes to metabolic remodeling in mice with MAFLD by modulating GM composition and serum amino acid metabolism (phenylalanine and tyrosine) [121].

FA and related polyphenols also contribute to lipid metabolic reprogramming and restoration of insulin sensitivity. These compounds enhance L-type Ca^2+^ channel-mediated Ca^2+^ influx, thereby improving insulin secretion [122]. FA directly targets the glucagon receptor (GCGR) and modulates PPARα and AMPK signaling, thereby suppressing lipotoxic apoptosis, mitochondrial dysfunction, and maladaptive substrate utilization in cardiomyocytes and ultimately ameliorating diabetic cardiomyopathy [123]. In parallel, FA promotes IRS-1 Tyr phosphorylation while inhibiting IRS-1 Ser phosphorylation and activates PI3K/Akt signaling to drive GSK3β phosphorylation. These effects facilitate GLUT4 translocation to the plasma membrane, restore insulin sensitivity, enhance glucose utilization, and support energy metabolism [124]. Integrated network pharmacology and metabolomics analyses suggest that FA maintains metabolic homeostasis by regulating key lipid metabolic pathways, including linoleic acid, arachidonic acid, and ether lipid metabolism, thereby exerting lipid-lowering and antihyperlipidemic effects [86]. Fatty acid synthase (FASN) catalyzes de novo fatty acid synthesis from acetyl-CoA and malonyl-CoA. FA alleviates iron overload–induced disturbances in hepatic lipid and bile acid metabolism by inhibiting FASN and CYP7A1, promoting fatty acid oxidation, and enhancing bile acid excretion. These effects suggest potential utility in preventing or treating related chronic liver diseases [125].

Regarding lipotoxicity and protection of β cells and adipose tissue, FA inhibits fatty acid binding to FABP3, thereby reducing free fatty acid influx into pancreatic islet β cells and limiting excessive β-oxidation under high-glucose/high-fat conditions. These effects may alleviate β-cell lipotoxic injury. Maternal FA supplementation improves offspring hyperglycemia, dyslipidemia, and islet structural abnormalities induced by a high-fat, high-sugar diet, suggesting a potential role in modulating maternal–fetal metabolic programming [126].

Glycosidic constituents, such as astragaloside IV, PF, and AMY, may exert anti-atherosclerotic effects via the JAK/STAT axis, which links inflammatory signaling to lipid metabolism. These compounds reduce JAK2, STAT1, and STAT3 phosphorylation, suppress JAK/STAT-mediated pro-inflammatory signaling, and improve lipid metabolism, thereby alleviating atherosclerotic burden [127]. Consistent with these effects, PF treatment lowers serum lipid levels and inflammatory cytokines and ameliorates hepatic steatosis [128]. Moreover, PF activates the farnesoid X receptor (FXR), upregulates hepatic efflux transporters, and downregulates uptake transporters and the bile acid-synthesizing enzyme CYP7A1, thereby restoring bile acid homeostasis within the gut–liver axis [129]. In addition, PF inhibits PI3K/Akt/FOXO1 and JAK2/STAT3 signaling, thereby reducing lipogenesis and pro-inflammatory cytokine expression [130]. PF also activates LXRα signaling, promotes cholesterol efflux from macrophages, and enhances reverse cholesterol transport. These effects are associated with increased HDL-C and ApoA-I levels, reduced TNF-α and MCP-1 levels and oxidative stress, and improved hepatic lipid metabolism and vascular function [131]. Regarding hepatic microcirculation and bile excretion, PF upregulates Nrf2 and Ntcp expression, suppresses NOX4 [132].

Animal studies further suggest that PF downregulates p-MYPT1, SREBP-1c, and FAS, thereby inhibiting fatty acid synthesis, while upregulating p-AMPK and LDLR to promote cholesterol uptake and maintain energy metabolic balance. Collectively, these changes are associated with reduced atherosclerotic plaque formation and an improved lipid profile [133]. Moreover, PF enhances estradiol secretion from peripheral adipose tissue, indirectly activates estrogen receptor-related signaling, and regulates glucose transport and energy metabolic homeostasis, thereby helping to alleviate the glucose metabolism abnormalities associated with ovarian functional decline [134].

CPBEB acts on multiple critical nodes, including the AMPK/SREBP–PCSK9/LDLR axis, the GCGR–PPARα–AMPK axis, the IRS-1/PI3K/Akt/GLUT4 axis, FXR/LXRα-mediated bile acid and cholesterol cycling, and JAK/STAT-coupled inflammatory metabolism. Collectively, these nodes establish an integrated metabolic regulatory network that coordinates lipid remodeling, restores insulin sensitivity, attenuates lipotoxicity, and maintains liver–gut axis function and bile acid homeostasis.

#### 4.3.5. Regulation of Platelet Activation and Coagulation

In platelet activation, phosphorylation of SRC at Tyr418 activates Src signaling, which recruits and activates phospholipase Cγ2 (PLCγ2) and protein kinase C (PKC), promoting granule release and increasing intracellular Ca^2+^ concentrations. These events trigger and amplify GPIIb/IIIa signaling, ultimately leading to platelet aggregation. HSYA upregulates *miR-9a-5p*, thereby inhibiting the SRC/PLCγ2/PKCδ pathway and reducing platelet activation and aggregation [135].

Regarding coagulation and thrombus formation, TMT-based quantitative proteomics suggests that AMY targets complement (C1s and C3) and coagulation-related pathways, thereby contributing to therapeutic effects [136]. In an arteriovenous shunt thrombosis model, FA has been reported to prolong coagulation time in mice. In rats, medium- and high-dose FA increases PT, APTT, and TT [137].

In AS, P2RY12 expression on VSMCs is upregulated and is predominantly localized near plaques and foam cells. P2RY12 activation suppresses autophagy, reduces cholesterol efflux, and promotes phenotypic conversion of VSMCs into foam-like cells with increased lipid deposition. Surface plasmon resonance analysis further suggests that ALB inhibits P2RY12 [138].

#### 4.3.6. Epigenetic Regulation

Regarding DNA damage and epigenetic regulation, CPBEB may act along a continuum from damage sensing to chromatin modification and cell fate determination. LSD1 primarily demethylates histone H3K4me1/2 or H3K9me1/2, thereby modulating transcription by altering chromatin structure and accessibility. Molecular docking combined with experimental validation suggests that LSD1 is a downstream target of ALB. ALB binds directly to LSD1 and inhibits its expression in a concentration-dependent manner. In turn, LSD1 overexpression attenuates the ability of ALB to suppress ferroptosis and inflammatory responses in microglia [139].

PF directly binds EZH2 and inhibits its methyltransferase activity, thereby reducing H3K27 trimethylation (H3K27me3) at the *PPARγ* promoter and relieving transcriptional repression of PPARγ. This, in turn, increases PPARγ expression and suppresses hepatic stellate cell activation and liver fibrosis progression [140]. In addition, PF inhibits *miR-29a* expression and restores NKRF activity, thereby blocking NF-κB/NLRP3 signaling, suppressing pro-inflammatory cytokine release, and restoring epithelial barrier integrity [141]. Hypoxic injury has been linked to hypertension, heart failure, and stroke. Under hypoxic conditions, the HIF-1α/*miR-210*/caspase-1/GSDMD pathway is activated, leading to increased IL-1β and IL-18 levels in brain tissue and enhanced astrocytic pyroptosis. PF reduces HIF-1α and *miR-210* expression, inhibits downstream caspase-1 and GSDMD activation, and decreases ROS production and LDH release, thereby improving cell viability and ameliorating tissue damage [142].

#### 4.3.7. Gut Microbiota Remodeling

HSYA targets hexokinase 1 (HK1), inhibits NLRP3/GSDMD-mediated pyroptosis, and shifts gut microbial composition by decreasing *Proteobacteria* and increasing *Bacteroidetes*, thereby establishing a gut–immune–metabolic interaction axis and conferring systemic protective effects [143]. Intervention with butyrate and FA increases IL-10 levels and the abundance of *Bifidobacterium pseudocatenulatum* while reducing jejunal IL-6, TLR4, and NF-κB (p65) expression, thereby conferring anti-inflammatory and immunoregulatory effects [144]. During in vitro fecal fermentation, FA enhances soluble fiber utilization, increases propionate and butyrate production, and reduces the abundance of *Streptococcaceae* and *Peptostreptococcaceae* [145]. In models of disordered lipid metabolism, FA promotes bile acid synthesis, suppresses lipogenesis-related gene expression, and restores β-oxidation-related gene expression. FA also increases the abundance of *Ruminococcaceae UCG-005* and *Lachnospiraceae* and lowers the *Firmicutes/Bacteroidetes* ratio, thereby attenuating oxidative stress, restoring GM homeostasis, and reducing lipid deposition [146]. AMY, in turn, modulates TLR4/NF-κB and MAPK signaling to inhibit inflammation and oxidative stress, reduces apoptosis and ferroptosis in intestinal epithelial cells, and improves GM composition [147].

Total paeony glycosides exert immunoregulatory and anti-inflammatory effects by modulating the GM–tryptophan metabolite–epithelial autophagy axis, with PF identified as a key active constituent [148]. PF increases GM abundance and diversity, improves amino acid metabolism, modulates the gut–brain axis, and confers anti-inflammatory and synaptoprotective effects [149]. PF activates ASIC3/ERK signaling to regulate intestinal fluid handling and visceral sensory nerve function, thereby improving intestinal motility disorders [150]. Moreover, PF promotes the secretion of growth arrest-specific protein 6 (Gas6) and phosphorylation of its receptor, Axl, thereby activating the Gas6/Axl/SOCS3 signaling axis. SOCS3 upregulation suppresses phosphorylated apoptosis signal-regulating kinase 1 (p-ASK1) and TF expression, attenuates inflammatory and coagulation responses, and ameliorates intestinal ischemia [151]. In models of AS and disordered lipid metabolism, PF downregulates TLR4 and NF-κB, reduces aortic plaque formation, reshapes linoleic acid and tryptophan metabolism and steroid biosynthesis, corrects abnormalities in galactose and glycerophospholipid metabolism, restores GM homeostasis and barrier function, and normalizes butyrate and propionate levels [128,152]. Multi-omics analyses further suggest that 11,12-epoxyeicosatrienoic acid (11,12-EET) is a key metabolite mediating the protective effects of PF-FMT. PF-FMT suppresses myocardial oxidative injury and iron-dependent cell death, thereby conferring cardioprotective effects [153].

In addition, PF improves intestinal barrier function, reduces epithelial permeability, and modulates the abundance of key genera, thereby inhibiting TLR4/MyD88/TRIF signaling and interrupting downstream inflammatory cascades. It also helps restore systemic immune homeostasis by decreasing the Th1/Th17 ratio and increasing the proportion of Treg cells [154]. PF also exhibits dual cholinesterase-inhibitory activity, suppressing AChE and BuChE activity in a dose-dependent manner. The maximal inhibition rates are 90.3% and 99.4%, respectively, with more pronounced inhibition of BuChE [155].

**Figure 3:**
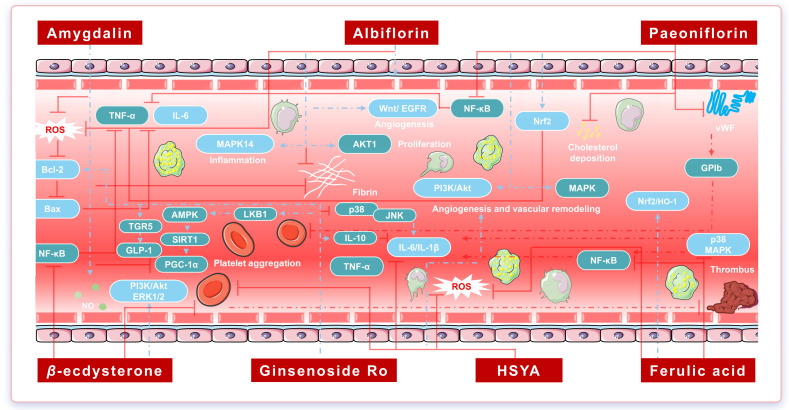
The pharmacological effects of the core component set for the promotion of blood circulation and the elimination of blood stasis. HSYA, Hydroxysafflor yellow A; ROS, Reactive oxygen species; Bcl-2, B-cell lymphoma 2; Bax, Bcl-2–associated X protein; TNF-α, Tumor necrosis factor alpha; IL-6, Interleukin-6; NF-κB, Nuclear Factor kappa B; TGR5, G protein–coupled bile acid receptor 1; GLP-1, Glucagon-like peptide-1; AMPK, AMP-activated protein kinase; SIRT1, Sirtuin 1; PGC-1α, Peroxisome proliferator-activated receptor gamma coactivator 1-alpha; PI3K, Phosphoinositide 3-kinase; Akt, RAC-alpha serine/threonine-protein kinase; ERK1/2, Extracellular signal-regulated kinase 1/2; MAPK14, Mitogen-activated protein kinase 14; LKB1, serine/threonine kinase 11; p38, p38 mitogen-activated protein kinase; JNK, c-Jun N-terminal kinase; EGFR, Epidermal growth factor receptor; PI3K, Phosphatidylinositol 3-kinase; Nrf2, Nuclear factor erythroid 2–related factor 2; HO-1, Heme oxygenase-1; GPIb, Glycoprotein Ib; vWF, von Willebrand factor.

Taken together, HSYA, FA, AMY, and PF remodel GM composition, regulate SCFA and fatty acid/bile acid metabolism, and coordinate TLR4/NF-κB and NLRP3/GSDMD signaling as well as T-cell subset profiles. These actions establish a gut–immune–metabolic interaction network linking intestinal microecology with innate and adaptive immunity, metabolic homeostasis, and cardio-cerebrovascular target organs (Figure 3).

### 4.4. Mechanisms of the Core Bioactive Component Set for the Regulation of Qi and the Relief of Pain

#### 4.4.1. Regulation of Disordered Qi Dynamics and Endothelial Dysfunction

Endothelial dysfunction (ED) is a key early event in the initiation and progression of AS. In an ox-LDL-induced HUVEC injury model, naringenin (NRG) increases cell viability, reduces lipid accumulation, and upregulates LC3-II expression. Further studies suggest that NRG downregulates key store-operated calcium entry (SOCE) proteins, STIM1 and ORAI1, thereby reducing intracellular Ca^2+^ influx. NRG attenuates ox-LDL-induced endothelial lipid deposition and cellular injury, thereby preserving endothelial homeostasis [156]. By activating AMPKα/Sirt1 signaling, NRG restores mitochondrial Ca^2+^ homeostasis and reduces ROS production. NRG also enhances eNOS activity, increases NO production, and improves endothelium-dependent vasodilation [157]. Furthermore, NRG activates endothelium-dependent NO/sGC/prostaglandin signaling, thereby promoting NO production, prostaglandin release, modulating Ca^2+^ and K^+^ channel activity. NRG inhibits voltage-operated calcium channel (VOCC)-mediated Ca^2+^ influx, reduces sarcoplasmic reticulum (SR) Ca^2+^ release, and decreases SERCA/SOCC-mediated Ca^2+^ reuptake. These changes ultimately lower intracellular Ca^2+^ levels and attenuate vascular smooth muscle contraction [158]. Platycodin D (PD) activates GPER-dependent Ca^2+^/CaMKKβ/AMPK and Ca^2+^/CaMKIIα signaling, enhances eNOS activity and NO production, and alleviates TNF-α-induced endothelial dysfunction [159].

Isoliquiritigenin (ISL) inhibits NOX2-mediated oxidative stress, downregulates inflammatory cytokine expression, and activates Nrf2/HO-1 antioxidant signaling, thereby improving endothelium-dependent vasorelaxation in the aortas of db/db mice [160]. ISL binds to SIRT6 (a class III histone deacetylase), promotes SIRT6 upregulation, and inhibits NLRP3 inflammasome activation and pyroptosis, thereby conferring protective and anti-inflammatory effects in vascular endothelial cells [161]. Neohesperidin (NHP) suppresses ROS generation and nucleic acid damage in HUVECs and blocks Ang II-induced VSMC migration and aberrant proliferation, thereby improving Ang II-induced hypertensive vascular remodeling and endothelial dysfunction [162].

NRG inhibits PI3K-mediated signaling and phosphodiesterase activity, thereby increasing intracellular cGMP levels and promoting VASP phosphorylation at Ser239. These changes suppress platelet α-granule secretion, fibrinogen binding, intracellular Ca^2+^ mobilization, and adhesion to collagen. NRG also inhibits platelet spreading and integrin outside-in signaling-driven clot retraction, ultimately restraining platelet activation [163]. In the coagulation system, protein disulfide isomerase (PDI) is released into plasma under cellular stress and contributes to thrombus formation. Naringin (NAR) binds PDI and modulates its conformation and activity, thereby delaying coagulation [164]. Formononetin (FNT) downregulates platelet surface CD36 expression and inhibits downstream ERK5 phosphorylation, thereby blocking CD36-mediated platelet activation and NET formation and ameliorating myocardial I/R injury [165].

Loss of semaphorin 3A (Sema3A) has been linked to EndMT in atrial fibrosis. NAR treatment upregulates Sema3A expression and downregulates EndMT-related factors, thereby ameliorating atrial fibrillation-associated pathology [166]. It also attenuates pulmonary arterial remodeling and dysfunction by inhibiting ERK/NF-κB signaling-mediated EndMT [167]. NAR suppresses VSMC proliferation, invasion, and migration, induces G1-phase cell-cycle arrest, and limits aberrant VSMC growth. It also reduces endothelial inflammation and apoptosis, lowers blood pressure, and enhances vascular protection, thereby exerting anti-AS effects [168]. In systemic blood pressure regulation, NAR inhibits renin–angiotensin system activation, reduces oxidative stress and inflammation, and modulates AT1R/PKC/NOX2/Raf-1/ERK1/2 signaling, thereby improving left ventricular and aortic dysfunction and structural remodeling in hypertensive rats [169]. Hesperidin (HSD) engages TRPV1-mediated Ca^2+^ signaling to initiate dual-axis regulation. It activates the CaMKII/p38 MAPK axis to enhance Mas receptor (MasR) expression and augments NO biosynthesis and vasodilation via the CaMKII/eNOS axis. The 3′-hydroxyl group on the flavonoid B ring and the twisted conformation are critical for TRPV1 activation [170].

A high-glucose environment activates endoplasmic reticulum (ER) stress, whereas NRG blocks nuclear colocalization of ATF4 and CHOP, suppresses ER stress-related apoptotic signaling, and improves insulin resistance. These effects reduce apoptosis and glycogen deposition, enhance glucose tolerance, and prevent high glucose-induced ER ultrastructural damage [171]. NRG also promotes angiogenesis in hypoxic HUVECs by modulating the *miR-223-3p*/IGF1R axis, thereby alleviating perfusion deficits associated with myocardial ischemia [172]. In addition, NRG downregulates VEGFA expression and inhibits PI3K/Akt/CREB5 signaling, thereby suppressing aberrant VSMC proliferation and migration and protecting against high glucose-induced injury [173]. Rhoifolin (ROF) downregulates MAPK8, TRAF6, and TRAF4 to inhibit stress and inflammatory signaling, while upregulating PDX1, SIRT1, INS1, and GLUT4. These coordinated effects support islet function and glucose uptake and enable bidirectional regulation of glucolipid metabolism and inflammatory responses [174]. HSD restores VEGFA/PI3K/AKT signaling, maintains cell-cycle homeostasis, and suppresses apoptosis and necrosis, thereby attenuating MAFLD progression [175].

At the molecular level, the regulate qi and relieve pain core component set engages multiple hubs, including the AMPKα/SIRT1–eNOS/NO–Ca^2+^ channel axis, the TRPV1–CaMKII–p38/eNOS pathway, the PDI–CD36–NETs axis, and EndMT/VSMC remodeling. Collectively, these hubs support an integrated mode of action spanning endothelial function, vascular tone, platelet activation, and vascular structural remodeling. These features support a modern pharmacological rationale for the TCM principle that regulating qi promotes blood flow and that free flow of qi relieves pain.

#### 4.4.2. Amelioration of Inflammatory Responses and Immune Dysregulation

NRG inhibits acidic sphingomyelinase (ASMase)-mediated lipid raft clustering and membrane recruitment of the NADPH oxidase subunit p47^phox^, thereby suppressing TLR4 membrane translocation and downstream NF-κB transcriptional activation. These effects ultimately attenuate vascular inflammation and macrophage activation [176]. Dietary NRG suppresses the aggregation and maturation of conventional dendritic cells (cDCs) and reduces CCL19 and its receptor CCR7 expression in the CNS, thereby limiting cDC chemotaxis and antigen-presenting capacity. NRG also inhibits pro-inflammatory cytokine secretion by splenic T cells and decreases infiltration of pathogenic T cells into the CNS [177]. During MAFLD progression, NRG inhibits TAK1 phosphorylation, downregulates JNK/ERK activation, reduces FOXO3a phosphorylation, and promotes FOXO3a nuclear localization, thereby enhancing transcription of apoptosis-related genes and inducing the programmed death of activated hepatic stellate cells (HSCs) [178].

By engaging the JAK/STAT axis and the cholinergic anti-inflammatory pathway, NAR suppresses p-JAK and p-STAT3 activation, thereby limiting M1 polarization and promoting an M2 phenotype, with potential neuroprotective and anti-inflammatory effects [179]. FNT activates α7nAChR and inhibits downstream JAK2/STAT3 signaling, thereby modulating macrophage polarization, attenuating inflammatory responses, and reducing lipid deposition [180]. In addition, FNT binds protein tyrosine phosphatase 1B (PTP1B) at the K116A site and inhibits its phosphatase activity, thereby maintaining STAT6 phosphorylation and promoting M2 polarization [181]. Furthermore, FNT targets GSK-3β and modulates the M1/M2 polarization balance in macrophages and microglia, thereby improving cardiac function and depression-like behaviors in mice with MI-associated depression [182]. Inhibition of the TLR4/NF-κB pathway further attenuates oxygen–glucose deprivation/reoxygenation-induced microglial inflammation and cellular injury [183].

Nobiletin (NOB) suppresses TLR4/NF-κB inflammatory signaling, activates Keap1–Nrf2 antioxidant signaling, and modulates IP3R-mediated Ca^2+^ homeostasis, thereby exerting multi-level protective effects against Aβ42-induced neuroinflammation and cellular injury [184]. Furthermore, NOB downregulates IL-6 expression and STAT3 phosphorylation while increasing nuclear FOXO3a phosphorylation, thereby engaging the IL-6/STAT3/FOXO3a axis, promoting macrophage autophagy, and suppressing inflammatory responses [185]. In addition, narirutin (NR) inhibits thioredoxin-interacting protein (TXNIP) expression and disrupts the interaction between NLRP3 and ASC, thereby reducing downstream pro-inflammatory cytokine release. NR also upregulates the tight junction proteins ZO-1 and occludin, thereby improving blood–brain barrier integrity after I/R injury [186,187]. Isoquercitrin targets the HSP90/SGT1/NLRP3 inflammasome pathway to suppress hepatic inflammatory responses, thereby exerting anti-steatohepatitis activity [188]. Moreover, isoquercitrin downregulates *miR-138-5p* and upregulates PGC-1α, thereby restoring lipid metabolic balance, reducing oxidative stress and inflammatory responses, and ameliorating hepatic injury in MAFLD [189]. PD activates PI3K/AKT signaling to improve cell survival and energy metabolism while suppressing excessive NLRP3 inflammasome activation and NF-κB-mediated inflammatory transcription. PD also attenuates ER stress (ERS)-mediated cellular inflammation and apoptosis, restores endothelial function, and improves early vascular inflammation and dyslipidemia in T2DM [190].

At the interface of metabolic inflammation and vascular/myocardial injury, NAR inhibits NF-κB-mediated inflammatory signaling and reduces IL-17 and caspase-3 expression, thereby attenuating cerebellar inflammation and tissue injury after cerebral I/R [191]. NAR reshapes macrophage metabolism and inflammatory responses by downregulating lipid uptake-related genes (*MSR1* and *CD36*) and upregulating cholesterol efflux genes (*ABCA1, ABCG1*, and *SR-B1*). These changes improve intracellular lipid handling and support its therapeutic potential in AS [192]. NAR exerts cardioprotective effects by activating *miR-126* and directly binding to and downregulating glycogen synthase kinase 3β (GSK-3β), thereby activating β-catenin signaling [193]. Furthermore, NAR activates the *PPARγ*/*miR-21* axis to drive macrophage polarization from an M1 to an M2 phenotype, thereby conferring anti-inflammatory and tissue-protective effects [194]. ROF improves glucose and lipid metabolic disorders by modulating the PPARα/SREBP1 axis and NF-κB inflammatory signaling, thereby attenuating hepatic injury and oxidative stress and ameliorating metabolic phenotypes in rats with T2DM [195]. Liquiritin (LIQ) inhibits NF-κB signaling, reduces inflammation and oxidative stress during lower-limb deep venous thrombosis formation, and thereby confers potential antithrombotic and vasoprotective effects [196]. In post-MI remodeling, LIQ suppresses CCL5/NF-κB signaling, thereby reducing oxidative stress and inflammation, improving cardiac function, and limiting myocardial fibrosis [197]. Moreover, LIQ activates AMPKα2 and inhibits the mTORC1/NF-κB axis, thereby attenuating LPS-induced septic myocardial inflammation, oxidative stress, and apoptosis and improving cardiac function and metabolic disturbances [198]. Liquiritigenin (LTG) inhibits TGF-β1 overexpression and downstream Smad2 phosphorylation, thereby limiting sustained inflammatory and fibrotic signaling and supporting its potential as a candidate agent for cardiovascular disease prevention and treatment [199].

CRQRP engages multiple key nodes, including TLR4/NF-κB, cGAS/STING, JAK/STAT, the NLRP3 inflammasome–α7nAChR/STAT6 module, and PPAR/miRNA axes. Collectively, these nodes establish a continuous regulatory spectrum spanning macrophage, dendritic cell, and microglial polarization, inflammasome activation, oxidative stress, and lipid/energy metabolic remodeling. This integrated network provides a mechanistic rationale for CRQRP’s anti-inflammatory, immune-reprogramming, and organ-protective effects in CVDs and related conditions.

#### 4.4.3. Regulation of Oxidative Stress and Mitochondrial Dysfunction

NRG activates the deacetylase SIRT1 and promotes deacetylation and upregulation of downstream targets, including FOXO3a and PGC-1α. These effects enhance SOD and CAT activity, promote mitochondrial biogenesis, reduce cellular and mitochondrial ROS, and attenuate endothelial senescence and oxidative stress [200]. In spontaneously hypertensive rats, NRG (100 mg/kg) lowers blood pressure, reduces thrombus formation, and restores KCl-induced aortic contractile responses. NRG also reduces lipid peroxide levels, normalizes glutathione and antioxidant enzyme activity, and mitigates myocardial necrosis and structural damage [201]. Compared with an ethanolic extract of *Citrus × paradisi* L. *peel*, NAR more effectively restores GSH levels and enhances CAT activity [202]. NAR confers protection by modulating the HO-1/GPX4 axis, restoring GSH and SOD levels, inhibiting ferroptosis, and suppressing pathological angiogenesis [203]. In addition, NAR has a strong iron-chelating capacity and reduces Aβ deposition by alleviating iron overload, thereby conferring antioxidant and neuroprotective effects [204]. LIQ pretreatment inhibits TNFR1/NF-κB/MMP9 signaling, increases mitochondrial membrane potential, and enhances SOD, CAT, and GSH-Px activity, thereby strengthening antioxidant defenses and alleviating hypoxia/reoxygenation-induced cardiomyocyte injury [205].

Under hypoxic stress that perturbs mitochondrial homeostasis, mild hypoxia suppresses SIRT1 nuclear translocation and BDNF release while enhancing NF-κB and caspase-1 activity. These changes are accompanied by reduced mitochondrial respiratory capacity and altered mitochondrial morphology. NRG administration restores mitochondrial respiration and structural integrity, partially reactivates the SIRT1/BDNF axis, and suppresses pro-inflammatory responses [206]. Furthermore, NAR activates SIRT1 and its downstream network, regulates the LKB1/AMPK/PGC-1α axis to promote mitochondrial biogenesis, activates FOXO1-dependent pathways to enhance autophagic capacity, and engages the Nrf2/NQO1 antioxidant pathway [207]. In murine and cellular models of septic cardiomyopathy, NRG inhibits CaMKII phosphorylation, downregulates Drp1 activation, and upregulates Bcl-2 expression, thereby restraining mitochondrial fission and reducing oxidative stress and apoptosis. These effects suggest coordinated regulation of mitochondrial homeostasis and electrical activity in cardiomyocytes [208]. NRG also activates the Nrf2/HO-1 pathway to suppress excessive ROS production and alleviate mitochondrial dysfunction, thereby attenuating NLRP3-mediated pyroptosis [209]. By activating the Nrf2/Keap1 axis in parallel with AMPK/Akt/mTOR signaling, NRG increases autophagic activity, downregulates α-SMA and collagen I expression, and slows disease progression [210].

Isoquercitrin confers protection by activating Nrf2/HO-1 signaling, inhibiting NLRP3 inflammasome activation, and restoring GM homeostasis [211]. NOB activates Nrf2 signaling to alleviate oxidative injury and modulates SREBP-1c and NF-κB signaling, thereby regulating lipid metabolism and suppressing inflammation [212]. HSD confers cardioprotection by modulating myocardial miRNA expression, activating Nrf2, and inhibiting NF-κB signaling [213]. LIQ activates the Keap1/Nrf2 antioxidant pathway, strengthens intracellular antioxidant defenses, and suppresses ER stress, thereby preserving blood–brain barrier structural and functional integrity [214]. ISL activates SIRT1/Nrf2 antioxidant signaling and promotes dissociation of the Keap1/Nrf2 complex, thereby enhancing Nrf2 nuclear translocation and binding to antioxidant response elements (AREs). This upregulates HO-1, GPX4, and SLC7A11; suppresses the pro-ferroptotic and mitochondria-injuring proteins ACSL4 and Drp1; promotes mitochondrial biogenesis; maintains balanced fusion–fission dynamics; and modulates mitophagy. Collectively, these actions restore cellular energy homeostasis, inhibit neuronal apoptosis, and attenuate myocardial mitochondrial structural damage and lipid peroxidation [215,216,217]. Furthermore, ISL suppresses ANXA2/STAT3/NF-κB inflammatory signaling and activates Nrf2-dependent antioxidant defenses while downregulating TAGLN2 [218]. ISL also regulates PI3K/Akt/mTOR signaling to promote hepatocyte autophagy, thereby reducing lipid deposition and inflammation [219].

NRG binds the F_1_F_0_-ATPase complex, a key mitochondrial membrane-associated energy-transducing enzyme, and selectively inhibits its ATP hydrolytic activity. Kinetic analyses indicate that NRG preferentially inhibits Ca^2+^-activated F_1_F_0_-ATPase, with weaker inhibition of the Mg^2+^-dependent form. This selectivity may help prevent mPTP opening, reduce ROS generation, and preserve mitochondrial function [220]. NOB restores high glucose-induced mitochondrial membrane potential disruption, activates mitophagy, and suppresses inflammatory signaling [221]. NAR alleviates intracellular Ca^2+^ overload and oxidative stress, strengthens antioxidant defenses, and reduces myocardial injury [222]. In myocardial I/R injury, NAR activates the IRF3/SLC7A11/GPX4 pathway and promotes mitochondrial translocation of NDUFS1, thereby attenuating ferroptosis in cardiac microvascular endothelial cells and improving coronary microcirculatory function [223]. At the single-cardiomyocyte level, 5 μM NAR enhances glibenclamide-sensitive KATP currents. In isolated hearts, this cardioprotective effect is attenuated when KATP channels are blocked by glibenclamide. In addition, NAR decreases Bax expression and reduces the Bax/Bcl-2 ratio, thereby protecting against myocardial I/R injury [224]. NAR also activates HIF-1α/BNIP3 signaling, promotes autophagic flux, and attenuates CoCl_2_-induced cardiomyocyte injury, supporting its potential as a natural modulator of autophagy for ischemic heart disease prevention and treatment [225].

NRG activates YAP, a key transcriptional coactivator in Hippo signaling, thereby inhibiting STAT3 phosphorylation, limiting ferroptosis and inflammatory amplification, and preserving mitochondrial function and epithelial barrier integrity [226]. NOB activates autophagy, as indicated by increased LC3-II levels and p62 degradation, while suppressing Hippo/YAP signaling and downregulating downstream target genes [227]. Isoquercitrin activates LKB1/AMPK signaling and increases phosphorylation of AMPK and its downstream target ACC, thereby modulating energy metabolism and alleviating oxidative stress. It also regulates YAP signaling, supporting antioxidant and hepatoprotective effects [228]. FNT directly binds YAP, prevents its nuclear translocation, and suppresses the transcription of Hippo pathway-dependent profibrotic genes, while also promoting mitochondrial biogenesis and metabolic reprogramming [229].

Under oxidative stress, HSD modulates the structure and function of ER–mitochondria contact sites, preserves mitochondrial homeostasis, and reduces ROS production, thereby alleviating intestinal barrier injury [230]. HSD also scavenges ROS, alleviates Ca^2+^ dyshomeostasis in the ER and mitochondria, and suppresses excessive MAPK activation [231]. Compared with quercetin, isoquercitrin exerts stronger antioxidant, anti-inflammatory, and cardioprotective effects in Ang II-induced cardiac injury, potentially via modulation of MAPK signaling and mitochondria-dependent apoptotic pathways [232]. NAR inhibits hyperactivation of the DRP1/LRRK2/MCU axis, thereby reducing mitochondrial Ca^2+^ overload, excessive fission, and aberrant mitophagy. In addition, NAR restores mitochondrial respiratory function in cardiomyocytes, modulates Ca^2+^-dependent mitochondrial permeability transition (MPT) pore kinetics, and attenuates oxidative stress-induced injury, thereby improving cardiac metabolic function [233].

Integrative metabolomics analyses identify glycolysis/gluconeogenesis, the pentose phosphate pathway, purine metabolism, and ascorbate and aldarate metabolism as key pathways associated with NAR-mediated attenuation of oxidative stress in hyperlipidemia and improved vascular endothelial function [234].

In addition, PD activates AMPK/PINK1/Parkin-mediated mitophagy and inhibits MAPK/NF-κB signaling, thereby attenuating oxidative stress and apoptosis under pathological conditions [235].

CRQRP engages multiple key nodes, including SIRT1, LKB1/AMPK, and PGC-1α; antioxidant axes such as Nrf2/Keap1 and HO-1/GPX4; regulators of mitochondrial dynamics and the mPTP; SLC7A11/GSH-dependent ferroptosis inhibition; and Hippo/YAP–autophagy–barrier function pathways. Collectively, these nodes constitute a continuum of regulation that links energy metabolism, oxidative stress, and mitochondrial homeostasis with ferroptosis and cell fate determination, thereby providing a unified mitochondrial framework for CRQRP’s actions in CVDs and related metabolic–inflammatory comorbidities.

#### 4.4.4. Reinforcement of Qi-Transforming Function in Energy and Metabolism

NRG confers cardioprotection against myocardial ischemia by reducing ROS and MDA levels, increasing antioxidant enzyme activity (GSH-Px, SOD, and CAT), and enhancing mitochondrial membrane potential and Ca^2+^-ATPase activity, thereby improving myocardial energy metabolism. NRG also suppresses L-type Ca^2+^ currents, cellular contractility, and Ca^2+^ transients, alleviates excessive Ca^2+^ signaling and Ca^2+^ overload, and inhibits cardiomyocyte apoptosis and inflammation [236]. Across the concentrations tested, NRG prolongs action potential duration (APD). With increasing pacing frequency, APD shortening shows sex-dependent differences; at 3 Hz, 50 μM NRG induces action potential and Ca^2+^ alternans in the female model [237]. NAR binds carbonic anhydrase II (CA-II) with high affinity and inhibits its enzymatic activity. It also downregulates NHE1, NCX1, and ACE1 expression, thereby attenuating Ang II-induced cardiomyocyte hypertrophy and ionic homeostasis disturbances [238].

In estrogen deficiency-induced obese rats, NRG upregulates genes related to mitochondrial biogenesis and white adipose tissue browning. It also activates the CaMKKβ/AMPK/ACC pathway to promote energy metabolism and mitochondrial biogenesis, thereby enhancing adipocyte metabolic activity [239,240]. In addition, selective activation of estrogen receptor β (ERβ) by NRG upregulates key β-cell transcription factors (*Pdx1* and *MafA*), thereby promoting insulin secretion, restoring β-cell function, and improving dysregulated glucose metabolism [241]. Furthermore, NRG activates AMPK, inhibits the acetyltransferase p300, reduces histone acetylation, and downregulates Txnip, thereby attenuating high glucose-induced β-cell stress and functional decline [242]. NRG activates SIRT1 signaling and promotes deacetylation of downstream targets (LKB1, PGC-1α, and NF-κB), thereby improving lipid metabolism, suppressing inflammation, and reducing oxidative stress [243]. NAR upregulates PPARα and carnitine palmitoyltransferase 1 (CPT1), enhances fatty acid β-oxidation, and promotes lipid catabolism [244]. NAR also activates TFEB-mediated lysosomal biogenesis and lipophagy, restores impaired autophagic flux, and attenuates hepatic lipid accumulation [245]. HSD activates SIRT1 and promotes PGC-1α deacetylation, thereby enhancing fatty acid β-oxidation, reducing hepatocellular lipid deposition, and ameliorating MAFLD [246]. HSD also activates AMPK and downregulates key lipogenic factors, thereby improving hepatic steatosis and metabolic disturbances [247]. Isoquercitrin targets key proteins (C-1-THF synthase, carbonyl reductase, and GSTP), activates AMPK, and inhibits SREBP-1c signaling, thereby regulating lipid synthesis and energy metabolism and supporting anti-obesity and anti-metabolic regulatory effects [248]. FNT activates SIRT1/PGC-1α/PPARα signaling, promotes fatty acid β-oxidation, and modulates hepatic lipid metabolism [249].

NAR increases the abundance of gut bacteria with bile salt hydrolase and 7α-dehydroxylase activity, thereby promoting conversion of cholesterol to bile acids. NAR further suppresses FXR/FGF15 signaling and upregulates CYP7A1 to enhance bile acid synthesis. It also downregulates PCSK9 and IDOL to facilitate reverse cholesterol transport, thereby attenuating AS [250]. ISL binds and inhibits the cholesterol transporter NPC1L1, thereby reducing cellular cholesterol absorption [251]. LIQ modulates Sirt1/FXR/Nrf2 signaling to improve bile acid transport, reduce oxidative stress and inflammation, and alleviate cholestatic liver injury [252]. NOB directly binds and activates PPARα, thereby upregulating lipid metabolism-related genes and attenuating cardiomyocyte hypertrophy [253]. NOB also modulates the PPARγ/CD36 axis to inhibit lipid uptake and foam cell formation in macrophages, thereby exerting anti-atherosclerotic effects [254]. Furthermore, orally administered NOB suppresses ANGPTL3 promoter activity and weakens LXRα-mediated transcriptional activation, thereby restoring lipoprotein lipase activity and lowering lipid levels [255]. NOB also upregulates hepatic CYP7A1 to promote bile acid synthesis and, by modulating the GM, alters bile acid composition in the ileum and feces [256].

NOB reshapes the exosomal miRNA profile by downregulating pro-adipogenic miRNAs (*miR-802-5p*) and upregulating energy metabolism-related miRNAs (*miR-574-5p*). These changes are associated with increased SIRT1 and FGF21 expression, reduced ACC, FASN, and CD36 protein levels, and enhanced lipolysis and energy metabolism [257]. Further studies show that *miR-433* directly targets and suppresses SIRT1 expression. NOB inhibits *miR-433*, thereby relieving repression of SIRT1 and activating SIRT1 signaling, which may improve cellular energy metabolism and anti-apoptotic capacity [258]. NOB also activates the ROR-mediated circadian regulatory axis, modulating the intrinsic adipocyte clock and downstream gene expression, thereby inhibiting adipogenesis, attenuating inflammation, and improving metabolic status [259]. In mice with liver-specific *Bmal1* deletion, NOB’s effects on cholesterol homeostasis depend on hepatic *Bmal1* [260]. NOB further modulates adiponectin–AdipoR1 signaling and inhibits gp91^phox^, thereby alleviating lipid accumulation and inflammatory fibrosis and improving MAFLD and systemic metabolic disturbances [261].

NOB and tangeretin migrate to the oil–water interface and alter the interfacial structure, thereby inhibiting enzymatic lipid hydrolysis. This interfacial migration mechanism appears to exert a stronger anti-lipolytic effect than direct lipase inhibition. Bile salts markedly enhance interfacial migration of NOB and tangeretin (6.80- and 12.31-fold, respectively), thereby indirectly suppressing lipid breakdown and absorption [262]. In addition, NOB interacts with bile salts at the oil–water interface, disrupts bile salt–stabilized lipid droplet emulsions, reduces interfacial stability, and inhibits free fatty acid release [263]. HSD reduces lipid droplet formation by ~35%, suppresses FASN and SCD1 expression, and upregulates SIRT1 and PGC-1α to promote fatty acid β-oxidation. Furthermore, HSD modulates key proteins (HK2, ENO1, and PI3K p110δ), thereby reshaping transcriptional programs related to insulin signaling and glucose metabolism and ameliorating HFD-induced insulin resistance and metabolic disturbances [264]. NAR competitively binds the catalytic site of pancreatic lipase and induces conformational changes that inhibit enzyme activity, thereby reducing lipid digestion and absorption and limiting fat accumulation [265]. LTG activates mTOR to suppress autophagy and lipogenic gene expression, thereby reducing lipid droplet accumulation. It also modulates PI3K/Akt signaling to improve glucose–lipid metabolism and mitochondrial energy balance [266,267]. Isoquercitrin attenuates NASH progression by inhibiting galectin-3-mediated insulin resistance and regulating mitochondrial lipid metabolic pathways [268]. ISL binds and stabilizes glycogen phosphorylase L (PYGL), thereby modulating lactate metabolism in cardiomyocytes, promoting lactate production, and ultimately improving myocardial energy metabolism and cardiac function [269]. In addition, ISL activates the IQGAP2/CREB/SIRT1 axis, thereby enhancing PPARα-dependent fatty acid oxidation and suppressing SREBP-mediated lipogenesis [270]. ISL also inhibits JNK signaling and promotes beige/brown transdifferentiation of hASC-derived white adipocytes. It upregulates UCP1 and PPARGC1α, modulates the TCA cycle, OXPHOS, and fatty acid metabolism, increases energy expenditure, and alleviates HFD-induced obesity [271,272]. PD promotes AMPK phosphorylation and downregulates PCK1 and G6Pase expression, thereby regulating hepatic gluconeogenesis and lipid metabolism and improving HFD/streptozotocin-induced metabolic dysfunction and liver injury [273]. Saikosaponin A (SSa) and saikosaponin D enhance AMPK/ACC phosphorylation and inhibit ERK1/2 and p38 MAPK signaling, thereby suppressing early adipocyte differentiation and lipogenesis [274]. Moreover, NRG activates the protein kinase A (PKA)/p38 MAPK axis and subsequently activates PPARγ, thereby potentiating isoproterenol (a β-adrenergic agonist)-induced thermogenesis and adipose browning [275].

CRQRP orchestrates a multiorgan remodeling program across the myocardium, adipose tissue, liver, and gut. This program is supported by three interconnected modules: (i) the SIRT1/AMPK/PGC-1α–PPAR axis; (ii) the cholesterol–bile acid–FXR/FGF15–CYP7A1 pathway; and (iii) a NOB-driven ROR/Bmal1–SIRT1/FGF21–exosomal miRNA–GLP-1 rhythmic network. Collectively, these modules reshape fatty acid β-oxidation, lipid droplet mobilization, cholesterol efflux, gut hormone rhythmicity, and energy allocation.

#### 4.4.5. Modulation of Neurotransmitter Signaling and Neurofunctional Network

MAO, a key enzyme in monoamine neurotransmitter metabolism, shows subtype-specific distribution in colonic tissue. NRG exhibits a threshold-dependent effect on MAO-A/B: at 25–300 mg/kg, it preferentially inhibits MAO-A, reduces 5-HT degradation, increases colonic 5-HT levels, and enhances intestinal peristalsis; at ≥150 mg/kg, it also inhibits MAO-B, which may increase dopamine accumulation and suppress colonic motility [276]. NOB activates the GDNF/AKT/FOXO3a/P21 pathway, promotes enteric neuronal survival and regeneration, and inhibits apoptosis, thereby protecting against HFD-induced enteric neuropathy and improving gut motility [277].

In rat models of vascular dementia, NRG upregulates synaptic proteins (SYP and PSD95) and NMDAR subunits (NR1 and NR2B), thereby enhancing synaptic plasticity and neural signal transmission and improving learning and memory deficits [278]. Combined treatment with PF and LIQ not only regulates lipid metabolism but also induces metabolic reprogramming and suppresses aberrant Ca^2+^ signaling, thereby alleviating neuropathic pain [279]. ISL activates GABA_B receptors and reduces evoked glutamate release at nerve terminals by the Gβγ-subunit-mediated inhibition of P/Q-type calcium channels and reduction of PKC phosphorylation, thereby attenuating excitotoxicity [280]. Moreover, ISL prevents Ca^2+^ dysregulation, preserves mitochondrial function, and supports cellular respiratory metabolism, thereby limiting glutamate excitotoxicity-induced neuronal death [281].

LIQ activates the cAMP/PKA/LKB1/AMPKα2 pathway and suppresses mTORC1 signaling and attenuates cardiomyocyte hypertrophy, thereby attenuating pressure overload-induced cardiac remodeling and dysfunction [282]. LTG improves mitochondrial function and inhibits L-type Ca^2+^ channel currents, thereby reducing ischemia-induced cardiomyocyte injury and Ca^2+^ dyshomeostasis [283]. Isoquercitrin suppresses the Piezo1-mediated NLRP3 inflammasome pathway, thereby mitigating neuroinflammation and tissue injury after intracerebral hemorrhage [284].

CRQRP engages multiple pathways involving nAChRs and MAO, 5-HT/DA and GABA receptors, and Ca^2+^-permeable mechanosensitive channels, thereby integrating central, enteric, and cardiovascular networks. Collectively, these actions may modulate gut motility, support emotional homeostasis, attenuate pain sensitivity, and protect the heart and brain.

#### 4.4.6. Improvement of Gut Microecology

NRG and NAR show bidirectional interactions with the GM. On the one hand, gut microbes metabolize these flavonoids into phenolic derivatives and ring-cleavage metabolites with distinct bioactivities. On the other hand, NRG and NAR remodel community composition and metabolite profiles, increasing probiotic taxa and reducing potential pathogens, thereby improving barrier integrity and metabolic homeostasis. NAR activates thermogenesis in brown adipose tissue (BAT), promotes adipose browning, and modulates the GM–adipose axis, thereby ameliorating HFD-induced metabolic dysfunction [285]. NRG preserves tight junction protein expression and localization (ZO-1, occludin, and claudin-5) and suppresses MLCK/p-MLC-mediated disruption of the gut–vascular barrier (GVB) and activation of the TLR4/NF-κB/NLRP3 pathway [286]. In HFD-induced obese mice, NRG attenuates weight gain, increases energy expenditure, and upregulates thermogenic and browning-related genes in inguinal white adipose tissue. In parallel, it improves gut dysbiosis and increases SCFA levels—particularly acetate—in the cecum and serum [287]. NRG also upregulates the stem cell factor (SCF)/c-Kit axis, increases beneficial taxa (*Planococcaceae*, *Bacteroides acidifaciens*, and *Clostridia_UCG-014*), and reduces Staphylococcus abundance, thereby preserving intestinal pacemaker cell function and coordinated smooth muscle contraction [288].

Dietary NOB supplementation reshapes GM community composition, improves SCFA and BA metabolism, strengthens barrier integrity, and reduces inflammation, thereby alleviating antibiotic-associated dysbiosis [289]. In HFD-fed mice, long-term NOB administration remodels GM structure, enhances demethylation capacity, and increases SCFA production [290]. Integrating untargeted metabolomics, studies indicate that NOB alleviates hepatic lipid accumulation by reshaping gut microecology and modulating key metabolic pathways, including myristoleic acid metabolism [291]. NOB also activates TFEB-mediated lysosomal biogenesis and lipophagy and inhibits inflammasome activation and maladaptive immune polarization, thereby attenuating MAFLD progression [292]. Moreover, by restoring GM diversity, NOB increases the abundance of BSH-positive taxa, promotes bile acid excretion, and suppresses FXR/SHP signaling, thereby improving hepatic cholesterol metabolism and inflammatory status and slowing MAFLD progression [293]. NOB also modulates MHC class II-mediated immune signaling, restores epithelial tight junction integrity and lipid metabolic homeostasis, and ameliorates HFD-induced intestinal barrier injury [294].

HSD reshapes GM composition by increasing the abundance of *Verrucomicrobia* and *Bacteroidota*. It also reduces the fecal levels of the branched-chain amino acids valine, leucine, and isoleucine by 27.4%, 50.1%, and 40.8%, respectively, thereby attenuating AS progression [295]. Liver metabolomics further indicates that HSD promotes arginine metabolism and pathways related to mitochondrial oxidative metabolism, which are associated with shifts in specific bacterial taxa [296]. NAR maintains intestinal epithelial integrity by inhibiting TLR4/p38 MAPK/NF-κB signaling, activating the Nrf2 antioxidant cascade, and strengthening tight junction integrity. In addition, NAR suppresses excessive cGAS/STING activation and exerts anti-inflammatory, antioxidant, and anti-apoptotic effects in intestinal I/R injury [297]. NR upregulates claudin-3, occludin, and ZO-1 and inhibits excessive TLR4/MAPK/NF-κB activation, thereby reducing pro-inflammatory cytokine levels and remodeling GM composition [298]. Isoquercitrin suppresses TLR4/MyD88/NF-κB signaling, reduces the expression and plasma levels of pro-inflammatory cytokines (IL-6, IL-1β, and TNF-α), and improves microbial composition, thereby alleviating LPS-induced mucosal barrier disruption [299].

Molecular interaction studies indicate that NOB directly binds HIF-1α and modulates downstream signaling, thereby suppressing iron-dependent lipid peroxidation and cell death. NOB also increases beneficial taxa and reduces potential pathogens, thereby restoring colonic barrier function and attenuating hepatic lipid accumulation [300]. In ApoE^−/−^ mice, isoquercitrin enriches β-glucosidase-active taxa (*Lactobacillus*, *Dubosiella*, and *Eubacterium_R*), increases indole metabolites (indolepyruvic acid, tryptophol, and 5-methoxyindoleacetic acid), and suppresses kynurenine pathway activity, thereby strengthening the intestinal barrier and reducing systemic inflammation [301]. ROF modulates GM diversity, restores Th17/Treg balance, and suppresses IL-17 signaling [302]. Moreover, ISL improves metabolic syndrome-related phenotypes by remodeling GM composition, strengthening the intestinal mucosal barrier, and suppressing adipose inflammation and insulin resistance [303]. FNT increases the abundance of taxa with anti-inflammatory associations (*Ligilactobacillus*, *Coprococcus*, *Blautia*, and *Muribaculaceae*) and reduces pro-inflammatory-associated taxa (*Treponema* and *Campylobacterota*), thereby suppressing inflammation and improving myocardial no-reflow and cardiac function [304]. FNT also increases SCFA-producing taxa, modulates inflammatory signaling, and upregulates Muc2 and occludin expression, thereby protecting the mucosal barrier [305].

SSa reshapes GM composition and downregulates intestinal FXR and its downstream target FGF19 at the mRNA level, thereby relieving negative feedback on intrahepatic bile acid synthesis and promoting cholesterol catabolism. SSa also suppresses the expression of the bile acid transporter ASBT, reduces intestinal bile acid reabsorption, and thereby improves hepatic bile acid homeostasis [306]. Under inflammatory conditions, SSa restores sterol homeostasis, increases 25-hydroxycholesterol (25-OHC) production, and inhibits NLRP3 inflammasome-mediated IL-1β release from macrophages [307].

**Figure 4:**
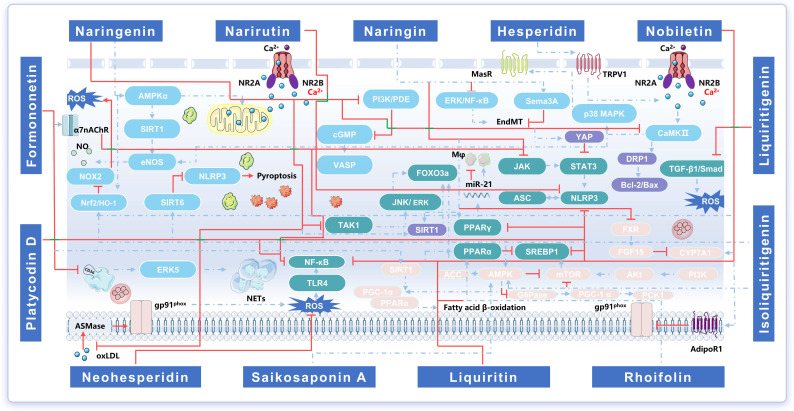
The pharmacological effects of the core component set for the regulation of qi and the relief of pain. ROS, Reactive oxygen species; α7nAChR, alpha-7 nicotinic acetylcholine receptor; NOX2, NADPH oxidase 2; Nrf2, Nuclear factor erythroid 2–related factor 2; HO-1, Heme oxygenase-1; AMPKα, AMP-activated protein kinase catalytic subunit alpha; SIRT1, Sirtuin 1; eNOS, Endothelial nitric oxide synthase; NLRP3, NOD-like receptor family pyrin domain containing 3; ERK5, Extracellular signal-regulated kinase 5; PI3K, Phosphoinositide 3-kinase; PDE, Phosphodiesterase; cGMP, Cyclic guanosine monophosphate; VASP, Vasodilator-stimulated phosphoprotein; TAK1, Mitogen-activated protein kinase kinase kinase 7; NF-κB, Nuclear Factor kappa B; TLR, Toll-like receptor 4; FOXO3a, Forkhead box O3a; PGC-1α, Peroxisome proliferator-activated receptor gamma coactivator 1-alpha; PPARα, Peroxisome proliferator-activated receptor alpha; Sema3A, Semaphorin 3A; EndMT, Endothelial–mesenchymal transition; JAK, Janus kinase; ASC, Apoptosis-associated speck-like protein containing a CARD; SREBP1, Sterol regulatory element-binding protein 1; p38 MAPK, p38 mitogen-activated protein kinase; YAP, Yes-associated protein; STAT3, Signal transducer and activator of transcription 3; CaMK II, Calcium/calmodulin-dependent protein kinase II; DRP1, Dynamin 1-like protein; Bcl-2, B-cell lymphoma 2; Bax, Bcl-2–associated X protein; TGF-β1, Transforming growth factor-beta 1; FXR, Farnesoid X receptor; FGF15, Fibroblast growth factor 15; CYP7A1, Cytochrome P450 family 7 subfamily A member 1; Akt, RAC-alpha serine/threonine-protein kinase; mTOR, Mechanistic target of rapamycin; AMPK, AMP-activated protein kinase; ACC, Acetyl-CoA carboxylase; G6Pase, Glucose-6-phosphatase; PGC-1 α, Peroxisome proliferator-activated receptor gamma coactivator 1-alpha; PCK1, Phosphoenolpyruvate carboxykinase 1; AdipoR1, Adiponectin receptor 1.

CRQRP remodels the GM, optimizes SCFA/BA and lipid metabolism, strengthens epithelial and vascular barrier integrity, suppresses TLR4/NF-κB, cGAS/STING, and NLRP3 signaling, and modulates ferroptosis and immune homeostasis. Together, these coordinated actions support the integrated regulation of metabolic homeostasis, inflammatory control, and barrier repair, thereby providing a mechanistic rationale for the potential long-term preventive and therapeutic roles of CRQRP in CVDs and related metabolic comorbidities (Figure 4).

### 4.5. An Integrated Analysis of the XFZYD Core Component Sets

The CPBEB, represented by HSYA, GRo, FA, *β*-ECD, AMY, ALB, and PF, primarily operates along a blood–vessel–stasis–metabolism axis. Mechanistically, CPBEB converges on ox-LDL handling, eNOS/NO signaling, EndMT–VSMC remodeling, and inflammatory pathways including NF-κB/MAPK, JAK2/STAT3, and NLRP3. It also engages metabolic stress–response programs, notably the SIRT1/AMPK–PGC-1α axis and antioxidant pathways centered on Nrf2/HO-1 and GPX4 that coordinate mitochondrial homeostasis and ferroptosis regulation. Through these interconnected networks, CPBEB coordinately modulates endothelial dysfunction, vascular remodeling, chronic inflammation and immune imbalance, lipid metabolic disturbances, and platelet/coagulation abnormalities (Table 2).

By contrast, CRQRP, represented by LIQ, NR, NAR, ROF, HSD, NHP, LTG, NRG, ISL, FNT, NOB, PD, and SSa, primarily modulates qi dynamics, reflected in the coordinated regulation of energy metabolism, neuroimmune and neuroendocrine signaling, and gut functions. Mechanistically, CRQRP engages key nodes, including AMPKα/SIRT1 and eNOS/NO signaling, Ca^2+^ channels and TRPV1–CaMKII–p38/eNOS pathways, inflammatory modules (TLR4/NF-κB, cGAS/STING, JAK/STAT, and NLRP3), metabolic regulators (SIRT1/AMPK/PGC-1α and PPAR), and Hippo/YAP–autophagy programs that support barrier integrity. Through these coordinated mechanisms, CRQRP regulates vascular tone and vasospasm, inflammation and immune polarization, oxidative stress and mitochondrial homeostasis, fatty acid β-oxidation, and cholesterol/bile acid metabolism. It also modulates nAChR/MAO and 5-HT/DA–GABA neurotransmitter systems, together with mechanosensitive channels, along the gut–cardiovascular axis (Table 2).

**Figure 5:**
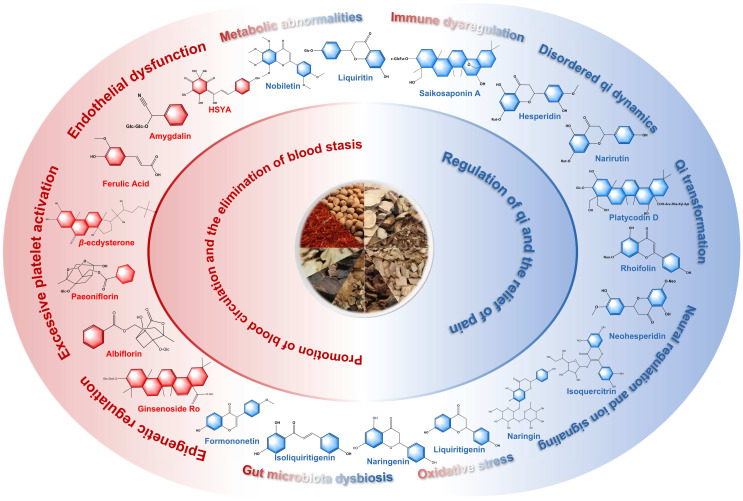
An integrated analysis of the XFZYD core component sets.

In summary, CPBEB primarily targets blood flow and the vascular wall to resolve stasis, whereas CRQRP emphasizes restoration of qi dynamics and energy metabolism to alleviate pain and functional impairment. These two sets intersect and complement each other across endothelial function, inflammation, mitochondrial biology, metabolism, together constituting a multi-target, integrated intervention framework through which XFZYD may benefit CVDs associated with a blood stasis pattern (Figure 5).

## 5. Biosafety of the Core Component Set

In Caco-2 cells, FA mitigates aflatoxin B_1_- and ochratoxin A-induced oxidative stress by inhibiting lipid and protein oxidation, reducing 4-HNE levels, scavenging ROS and preserving thiol groups, increasing SOD activity, restoring mitochondrial function, and decreasing Nrf2 ubiquitination [308,309].

AMY can be metabolized via both cyanogenic and non-cyanogenic pathways, and its metabolic efficiency in the GM is higher than that in liver microsomes. The GM plays a dual role in AMY metabolism in vivo. Taxa enriched in *sulfurtransferases*—including *Coriobacteriaceae bacterium*, *Butyricicoccus porcorum*, and *Akkermansia muciniphila*—may contribute to detoxification, whereas *Bifidobacterium pseudolongum*, *Marvinbryantia formatexigens*, and *Bacteroides fragilis* are associated with cyanide generation [310].

The peony glycosides ALB and PF protect against acetaminophen- and toxic herbal constituent-induced acute liver injury by inhibiting pyroptosis, limiting HMGB1 release, and suppressing CYP2E1/JNK signaling [311,312]. In addition, ALB and PF promote autophagy via the SIRT1/mTOR/TFEB pathway and suppress NLRP3-mediated pyroptosis, thereby attenuating liver toxicity associated with ZnO nanoparticles and other emerging materials [313]. PF is a P-gp substrate and a moderate inhibitor of *CYP1A2*, *CYP2C11*, and *CYP3A1*, suggesting potential drug–drug interactions with co-administered agents metabolized by these pathways [314].

NRG modulates the Bcl-2/Bax balance and caspase-3/9 activation and concurrently suppresses lipid peroxidation and inflammatory mediator production, thereby protecting against drug-, heavy metal-, and mycotoxin-induced injury in the liver, kidney, and other organs [315]. NRG also inhibits multiple CYP isoforms (CYP2E1, CYP3A4, CYP2C9, and CYP2D6), which may slow the clearance of co-administered drugs and increase the potential for drug–drug interactions—a hepatoprotective/nephroprotective yet potentially interacting double-edged sword [316]. In rats, NRG (100 mg/kg/day) significantly reduced 5-fluorouracil (5-FU)-induced increases in blood urea nitrogen (BUN), MDA, TNF-α, and caspase-3, decreased renal cell apoptosis, and attenuated nephrotoxicity [317]. NRG also decreases NO levels, enhances antioxidant enzyme activity, restores the GSH/GSSG balance, and downregulates caspase-3 expression, thereby counteracting aluminum nanoparticle-induced hepatic and renal toxicity [318].

NOB modulates aryl hydrocarbon receptor (AhR)/CYP1 signaling, thereby reducing B[a]P biotransformation, decreasing DNA adduct formation from B[a]P diol epoxide metabolites, and attenuating cytotoxicity and DNA damage [319]. A solid-dispersion NOB nanodelivery system was developed using the amphiphilic block copolymer Soluplus and PVP/VA64 as carriers, which increased NOB bioavailability. This formulation also alleviated acetaminophen (APAP)-induced liver dysfunction and histopathological injury, at least in part by activating Nrf2 signaling [320].

HSD targets VDAC3 to regulate mitochondrial function, suppress ferroptosis and necrotic cell death, and limit mitochondrial injury mediated by the ROS/p53/PGC-1α axis. These actions collectively block mtROS-driven PANoptosis and attenuate deoxynivalenol-induced hepatic and renal dysfunction [321,322]. HSD also modulates mitochondria-associated ER membrane (MAM) architecture and IP3R–MCU Ca^2+^ signaling, thereby preventing mitochondrial Ca^2+^ overload and oxidative stress and improving mitochondrial function. These effects may alleviate intestinal injury and provide mechanistic support for HSD in preventing or treating mycotoxin-related gut damage [323]. In mice with acetaminophen intoxication, HSD nanoparticles improved serum lipid profiles and liver function markers, enhanced antioxidant defenses, and downregulated TNF-α as well as CYP2E1 and CYP3A11 expression [324]. HSD nanocomposites mitigate doxorubicin-induced nephrotoxicity, potentially through integrated antioxidant, anti-inflammatory, and anti-apoptotic effects mediated by SIRT1/HIF-1α/VEGF/NF-κB signaling [325].

NAR exerts antioxidant, anti-inflammatory, and anti-apoptotic effects, potentially through an enoyl-CoA hydratase 1 (ECHS1)-related pathway, thereby providing mechanistic support for its potential use in preventing or treating anthracycline-induced cardiotoxicity [326]. NAR inhibits paraquat-induced epithelial–mesenchymal transition, migration, and invasion by suppressing the NDRG1/JNK axis and upregulating PPARγ signaling, thereby attenuating pulmonary inflammation and fibrosis [327]. NAR enhances antioxidant defenses, suppresses inflammatory signaling, and modulates AKT/PI3K/Smad3/Smad7 signaling, thereby alleviating digoxin-induced nephrotoxicity and oxidative stress-related injury [328].

NR potently inhibits OATP1A2- and OATP2B1-mediated transport, with IC_50_ values of 22.6 and 18.2 μM, respectively. Comparative analyses indicate that the inhibitory potency of three grapefruit-derived flavonoids follows the order NRG > NR > NAR, which may influence oral drug absorption and pharmacokinetics [329].

Isoquercitrin attenuates pirarubicin (THP)-induced myocardial oxidative stress and apoptosis by modulating Phlpp1/AKT/Bcl-2 signaling, stabilizing mitochondrial membrane integrity, and inhibiting cytochrome c release and apoptosome assembly [330]. Isoquercitrin may mitigate cardiotoxicity by downregulating immune hub genes (*CCL19* and *PADI4*), thereby suppressing doxorubicin-induced immune inflammation and oxidative stress [331].

LIQ attenuates cisplatin-induced hepatic inflammation, oxidative stress, and apoptosis by inhibiting p38 MAPK/p53 signaling, downregulating pro-apoptotic proteins (Bax, caspase-3, and PUMA), and upregulating Bcl-2 [332]. LTG activates the NRF2/SIRT3 axis, improves mitochondrial function, and suppresses oxidative stress and apoptosis, thereby alleviating cisplatin-induced acute kidney injury [333]. LTG also activates NRF2 signaling through both KEAP1-dependent and p62-mediated mechanisms, thereby enhancing cellular adaptive responses to oxidative stress and protecting against drug-induced liver injury [334]. ISL covalently modifies key KEAP1 cysteine residues (Cys226 and Cys288), activates Keap1/Nrf2/ARE signaling, and thereby enhances endogenous antioxidant defenses while reducing cisplatin-induced oxidative stress, mitochondrial damage, and hair cell apoptosis. These effects may protect against cisplatin-associated hearing loss [335]. AI-based deep learning combined with phenotypic screening identifies ISL as protective in anthracycline-induced cardiotoxicity models, potentially by reducing DNA double-strand breaks and oxidative damage and activating HO-1-mediated antioxidant defenses [336].

SSa inhibits integrin β3-mediated metastasis in triple-negative breast cancer and alleviates doxorubicin-induced cardiotoxicity. Moreover, co-delivery of SSa and doxorubicin via a nanodelivery system enhances chemotherapeutic efficacy and tumor targeting [337].

This core component set exhibits a coherent pattern along a detoxification–organ protection–drug metabolism axis. These constituents may serve as protective adjuncts for patients with CVDs who receive polypharmacy or chemotherapy, or who experience long-term toxicant exposure. However, their broad modulation of CYP enzymes, transporters, and the GM may increase the risk of drug–drug interactions and interindividual variability, underscoring the need for systematic pharmacokinetic and pharmacodynamic safety assessments during clinical translation.

## 6. Clinical Applications of the Core Component Set

Clinical trials and population-based studies suggest that, at specific doses and treatment durations, HSD is associated with improved fasting glucose, insulin levels, and lipid profiles, and may be accompanied by downregulation of inflammation-related transcriptional signatures [338,339,340,341,342]. Systematic reviews and multiple preclinical studies consistently indicate that NAR alleviates oxidative stress and inflammation, promotes tissue protection and functional recovery across diverse models, and shows a trend toward cardiovascular benefit [343]. Metabolomics analyses show that circulating ISL concentrations are inversely associated with MAFLD risk. When combined with metabolic and inflammatory indices, ISL may support the development of a noninvasive model with high predictive accuracy for MAFLD risk stratification and diagnosis [344].

## 7. Discussion

The onset and progression of CVDs involve multiple interacting processes, including endothelial dysfunction, chronic inflammation, immune imbalance, metabolic dysregulation, oxidative stress, and mitochondrial dysfunction; therefore, single-target interventions are generally insufficient to alter disease trajectories at the systems level. Clinically, standard regimens that combine antiplatelet agents, statins, ACE inhibitors/ARBs, and β-blockers exemplify a multi-target, combination-treatment paradigm. Traditional Chinese formulae likewise combine multiple herbs to achieve multi-component, multi-pathway synergy. Within the TCM framework, these prescriptions regulate qi and blood and coordinate zang-fu functions and meridian networks, a systems-oriented strategy that conceptually aligns with modern multi-target combination therapy for CVDs. Building on this rationale, we propose a “core set” concept: a subset of key constituents within a complex prescription that is expected to contribute disproportionately to a defined therapeutic function. We then systematically characterize its network pharmacology profile, pharmacokinetics, and pharmaceutic properties to clarify the material basis underlying XFZYD’s putative efficacy in CVDs.

This study identifies HSYA, GRo, FA, *β*-ECD, AMY, ALB, and PF as constituents of CPBEB. Network analyses indicate that this core set is enriched in pathways involved in lipid metabolism and AS, the AGE–RAGE signaling pathway in diabetic complications, relaxin signaling, and HIF-1 signaling. Collectively, these pathways may enable coordinated modulation of endothelial dysfunction, vascular smooth muscle cell proliferation and migration, inflammatory and immune activation, oxidative stress, and apoptosis—an effect pattern that aligns with the TCM notion of invigorating blood and resolving stasis. Compared with conventional single-compound-centric approaches, this blood-activating/stasis-resolving core set highlights complementary actions of multiple constituents at distinct nodes within a shared pathological network. This perspective may help explain how XFZYD exerts system-level effects in complex settings, including AS, I/R injury, and cardiometabolic complications.

In parallel, this study identifies LIQ, NR, NAR, ROF, HSD, NHP, LTG, NRG, ISL, FNT, NOB, PD, and SSa as constituents of CRQRP. Compared with the blood-activating/stasis-resolving core set, the qi-regulating/pain-relieving core set places greater emphasis on qi–blood coupling, particularly the linkage between disordered qi dynamics and endothelial dysfunction. Network analyses suggest that this core set is enriched in PI3K–Akt signaling, AGE–RAGE signaling in diabetic complications, nitrogen metabolism, and lipid metabolism and AS. These pathways converge on endothelial injury, inflammatory responses, immune polarization imbalance, disrupted energy and lipid metabolism, and oxidative stress. Notably, this core set engages key nodes—including TLR4/NF-κB, cGAS–STING, JAK/STAT, the NLRP3 inflammasome–α7nAChR/STAT6 module, and PPAR/miRNA axes—thereby delineating a continuous regulatory spectrum spanning macrophage, dendritic cell, and microglial polarization, inflammasome activation, ferroptosis, and mitochondrial remodeling. Together, these features provide a coherent mechanistic framework for interpreting the traditional actions of XFZYD—regulating qi to unblock the fu-organs, harmonizing qi to resolve stasis, and relieving pain to protect vital organs—in modern molecular terms.

Most constituents in the core set are highly polar and show limited oral absorption in their parent forms. Systemic exposure therefore depends largely on GM-mediated deglycosylation, followed by phase II conjugation (glucuronidation and sulfation). Renal and biliary excretion are the principal clearance routes. Accordingly, effective in vivo exposure is shaped not only by the intrinsic properties of individual compounds but also by multiple extrinsic factors. First, formula compatibility and co-administration with foods or concomitant drugs can markedly alter exposure metrics (AUC, C_max_, and MRT). These changes may arise from the modulation of transporters (P-gp) and metabolic enzymes (CYPs and UGTs). Second, pathological states (ischemia and diabetes) can alter hepatic–intestinal metabolic capacity and the availability of conjugation substrates. This may redirect metabolic fluxes and thereby lengthen or shorten the therapeutic window. Meanwhile, shifts in GM composition can influence deglycosylation efficiency and, in turn, the formation of absorbable species. For some constituents, circulating exposure may be dominated by metabolites rather than parent compounds. Overall, these considerations suggest several priorities for future research and translation. First, formulation and delivery strategies should be optimized to improve oral absorption and stabilize systemic exposure. Second, pharmacokinetic interactions arising from compatibility and combination therapy should be systematically evaluated. Third, safety and individualized-use frameworks should incorporate stratification by GM profiles and disease states.

To overcome key pharmaceutic bottlenecks of the core constituents—high polarity, poor solubility and permeability, extensive first-pass metabolism, and tissue-barrier constraints—recent research has pursued three complementary strategies. These include green, efficient extraction (deep eutectic solvents and ultrasound-assisted extraction), structural and phase-state modulation (solidified phospholipid technology and dual-lipid matrices), and multi-scale delivery systems (phospholipid complexes, nanostructured lipid carriers, micelles and liposomes, microcapsules, and targeted hydrogels). Collectively, these approaches can enhance systemic exposure and tissue targeting while preserving the spectrum of pharmacodynamically active substances. In addition, fermentation and synthetic biology platforms can enable efficient production of key metabolites (NRG), supporting scalable supply and downstream formulation development. Overall, these strategies provide a pragmatic pharmaceutic foundation for multi-component, multi-target, organ-level interventions in CVDs. However, systematic evidence is still needed on formulation consistency and quality control, in vivo distribution and long-term safety, interactions with concomitant drugs, and clinically oriented exposure–response validation. Addressing these gaps will be essential to translate precision delivery and exposure control into practice.

Nevertheless, several limitations should be acknowledged. Evidence supporting the proposed core constituent sets is derived mainly from heterogeneous preclinical in vivo and in vitro studies that differ in species, disease models, dosing regimens, routes of administration, and formulation types, which complicate direct translation across settings. In addition, the identification of the core sets is constrained by the available analytical coverage and the literature evidence, and contributions from other potentially important constituents—particularly those with low systemic exposure or that remain understudied—cannot be excluded. Moreover, potential antagonistic effects and non-linear interactions among constituents cannot be ruled out. Accordingly, the present work provides an evidence-weighted, functional-enrichment perspective rather than an exhaustive delineation of the formula’s complete pharmacodynamic material basis.

## 8. Limitations and Outlook

Despite the promising pharmacological profiles of the two core component sets, several limitations of XFZYD research should be acknowledged. First, the current evidence was mainly derived from preclinical studies and small-scale clinical trials. High-quality randomized controlled trials are still scarce. Secondly, as a multi-herb, multi-component formula, XFZYD is subject to variability in raw materials, processing, and dosage forms, which can complicate quality control, obscure exposure and dose relationships in vivo, and contribute to heterogeneity in clinical outcomes. Thirdly, because patients with CVDs frequently receive polypharmacy, potential herb–drug interactions and inter-individual variability in the pharmacokinetics require systematic evaluation. Thus, rigorous safety monitoring and interaction studies are needed when XFZYD is used in the clinic. In addition, decoction may pose practical problems for long-term use and may reduce adherence in real-world cardiovascular populations. Finally, standardized operational criteria for syndrome-guided applications of XFZYD require further refinement and validation.

Building on these findings, we propose a pharmacology-driven “core component set” framework that integrates quality biomarkers, in vivo exposure markers (i.e., blood- and intestine-exposed constituents), and clinically meaningful endpoints to advance XFZYD toward mechanism-informed and evidence-based clinical application. Accordingly, future work should prioritize: (i) establishing efficacy-oriented quality control for XFZYD and systematically evaluating the differences in XFZYD formulations in chemical composition, systemic exposure, efficacy, and safety; (ii) conducting rigorously designed clinical studies in targeted patients and systematically monitoring safety and herb–drug interactions when used in the clinic; (iii) developing and validating biomarkers by integrating multi-omics analyses with experimental and clinical validation; (iv) exploring the optimization of formulation and microbiome-oriented strategies to improve bioavailability, tissue delivery, and usability; however, their clinical value requires rigorous validation. In conclusion, realizing the future potential of XFZYD will require addressing issues related to quality consistency, efficacy, safety, and herb–drug interactions.

## 9. Conclusions

By emphasizing component synergy, integrating modern pharmaceutics with gut microecology, and synthesizing mechanistic evidence, this review indicates that XFZYD does not rely on a single marker compound to achieve its therapeutic effects. Rather, XFZYD acts through two core sets—one associated with promoting blood circulation and removing blood stasis and the other with regulating qi flow and relieving pain. Together, these sets form a complementary network spanning multiple targets, pathways, and organs, enabling the integrated modulation of CVD pathophysiology. This framework advances understanding of the material basis and mechanisms of classical formulae and provides a rationale and roadmap for developing multi-target cardiovascular agents, optimizing multi-component preparations, and advancing translational research along the formula–component–target–pathway–disease axis.

## Figures and Tables

**Table 1:** Core component sets of Xuefu Zhuyu Decoction.

Prescription	Core Component Sets	Herbs	Compounds	Formulae	Structure Types	Refs.
Xuefu Zhuyu Decoction	Core component set for promoting blood circulation and eliminating blood stasis	CARTHAMI FLOS	HSYA	C_27_H_32_O_16_	Flavonoids	[7]
		PERSICAE SEMEN	AMY	C_20_H_27_NO_11_	Others	[7]
		CHUANXIONG RHIZOMA & ANGELICAE SINENSIS RADIX	FA	C_10_H_10_O_4_	Phenolic acids	[7]
		ACHYRANTHIS BIDENTATAE RADIX	*β*-ECD	C_27_H_44_O_7_	Others	[7]
		PAEONIAE RADIX RUBRA	PF	C_23_H_28_O_11_	Monoterpenes glycosides	[7]
		PAEONIAE RADIX RUBRA	ALB	C_23_H_28_O_11_	Monoterpenes glycosides	[7]
		ACHYRANTHIS BIDENTATAE RADIX	GRo	C_48_H_76_O_19_	Triterpenoid saponins	[7]
	Core component set for regulating qi and relieving pain	BUPLEURI RADIX	SSa	C_42_H_68_O_13_	Triterpenoid saponins	[7]
		AURANTII FRUCTUS	HSD	C_28_H_34_O_15_	Flavonoids	[7]
		AURANTII FRUCTUS	NOB	C_21_H_22_O_8_	Flavonoids	[7]
		GLYCYRRHIZAE RADIX ET RHIZOMA	ISL	C_15_H_12_O_4_	Flavonoids	[7]
		GLYCYRRHIZAE RADIX ET RHIZOMA	LIQ	C_21_H_22_O_9_	Flavonoids	[7]
		GLYCYRRHIZAE RADIX ET RHIZOMA	LTG	C_15_H_12_O_4_	Flavonoids	[7]
		AURANTII FRUCTUS	ROF	C_27_H_30_O_14_	Flavonoids	[7]
		AURANTII FRUCTUS	NR	C_27_H_32_O_14_	Flavonoids	[7]
		AURANTII FRUCTUS	NHP	C_28_H_34_O_15_	Flavonoids	[7]
		PLATYCODONIS RADIX	PD	C_57_H_92_O_28_	Triterpenoid saponins	[7]
		GLYCYRRHIZAE RADIX ET RHIZOMA	FNT	C_16_H_12_O_4_	Flavonoids	[7]
		AURANTII FRUCTUS	NRG	C_15_H_12_O_5_	Flavonoids	[7]
		AURANTII FRUCTUS	NAR	C_27_H_32_O_14_	Flavonoids	[7]

**Table 2:** Overview of the core active component sets of Xuefu Zhuyu Decoction in cardiovascular diseases.

Core Bioactive Component Set	Pathophysiological Processes	Compounds	Mediators	Application	Refs.
Core component set for promoting blood circulation and eliminating blood stasis	Endothelial dysfunction and vascular structural abnormalities	HSYA	Piezo1, YAP, JNK; SphK1, S1PR3, RhoA, ROCK, F-actin, LC3, HIF-1, BNIP3, Notch1; HIF-1α, ZO-1; Src, MMP-9, P-gp;	In vivo	[8,9,10,11,12]
		FA	gp91^phox^, MMP-2; Nrf2, HO-1, NF-κB, TGF-β;	In vivo	[13,15]
		FA	L-type Ca^2+^ channels;	In vitro	[14]
		AMY	Mapk1, Mapk8, Mapk14, Fos, Jun, NF-κB p65;	In vivo	[17]
		AMY	NF-κB p50, TLR4, Bcl-2, Bax;	In vitro	[16]
		ALB	EGFR, STAT3, AKT1, VEGF-A; PARP1, NF-κB;	In vitro	[18,19]
		PF	VEGFA, MMP9, MMP2, ERα; RXRα;	In vivo	[20,21]
		PF	Nrf2, HO-1;	In vitro	[23]
	Chronic inflammatory responses and immune dysregulation	HSYA	p-p38, p-JNK; JAK2, STAT3, SOCS3; NLRP3, AIM2, Pyrin, Cleaved-Caspase1, GSDMD, ASC, HMGB1;	In vivo	[24,32,36]
		HSYA	PI3K, AKT, mTOR, NF-B; ZBP1, NLRP3; NLRP3, Caspase-1, GSDMD, GSDMD-N;	In vitro	[37,39,40]
		FA	IKKβ, NF-κB; ERK, MEK;	In vivo	[25,26]
		GRo	p38, JNK, Bcl-2	In vivo	[27]
		ALB	TLR4, NF-κB; P38MAPK; p65, IκBα, JNK; P2X7R, NURR1;	In vivo	[28,29,30,31,46]
		ALB	NLRP3, TCA cycle;	In vitro	[41]
		AMY	JAK2, STAT3;	In vivo	[33]
		PF	p-PI3K, p-Akt; TLR4, MYD88, NF-κB, NLRP3; P38 MAPK, ERK, mTOR; JNK1, JNK2;	In vivo	[45,47,48,49]
		PF	NLRP3;	In vitro	[42]
	Oxidative stress and mitochondrial dysfunction	HSYA	MDH1;	In vivo	[71]
		HSYA	HIF-1α, SLC7A11, GPX4; SF3A1; SIRT1, FOXO1, PGC1α;	In vitro	[51,70,73,74]
		FA	ERK, p-Akt, AChE, BACE; APE1; SIRT1, AMPK, SREBP1, ACC1; Bax, caspase3, caspase9, p53;	In vivo	[56,69,77,79]
		PF	Nrf2, HO-1, SLC7A11, GPX4; PKCδ, NF-κB; PI3K, AKT; NLRP3, GSDMD;	In vivo	[59,61,83,84,85,86]
		PF	SIRT1, PINK1, parkin; cytochrome c, caspase3, HDAC4; SOD, GSH-Px, CAT, IL-10;	In vitro	[75,80,82]
		*β*-ECD	Nrf2, HO-1, SLC7A11, GPX4; AKT, Nrf2;	In vivo	[59,61]
		*β*-ECD	Nrf2, HO-1, ARE; ASK1, p38 MAPK, p53; Bcl-2, Bax, JNK;	In vitro	[60,62,63]
		AMY	Nrf2, catalase, SOD-2, GPX-4;	In vitro	[64]
		ALB	PGK1, Nrf2, HO-1;	In vivo	[68]
		ALB	Akt, Nrf2, HO-1;	In vitro	[67]
		GRo	LKB1, AMPK, SIRT1;	In vitro	[76]
	Metabolic abnormalities and insulin resistance	HSYA	GIP, GIPR;	In vivo	[87]
		GRo	GLP-1, TGR5	In vivo	[88]
		FA	GCGR, PPARα, AMPK; PPARα, Acox1, Adipoq, Bsep, Shp, Fasn, Acc, Cyp7a1;	In vivo	[91,93]
		FA	L-type Ca^2+^ channel; IRS-1, Akt, AMPK;	In vitro	[90,92]
		PF	FXR, CYP7A1; PI3K, AKT, FoxO1, JAK2, STAT3; Nrf2, Ntcp, NOX4; ROCK, AMPK, SREBP-1c;	In vivo	[97,98,100,101]
	Coagulation and excessive platelet activation	HSYA	SRC, PLCγ2, PKCδ, MEK, ERK1/2;	In vivo	[103]
		FA	PT, APTT, TT	In vivo	[105]
		ALB	P2RY12;	In vitro	[106]
	Epigenetic regulation	ALB	LSD1;	In vitro	[107]
		PF	miR-29a, NKRF, NF-κB, NLRP3; HIF-1α, miR-210, caspase-1, GSDMD; EZH2, H3K27me3, PPARγ	In vivo	[108,109,110]
	Gut microbiota–immune–metabolic axis	HSYA	HK1, NLRP3, GSDMD;	In vivo	[111]
		FA	IL-6, TLR4, NF-κB (p65);	In vivo	[112]
		AMY	TLR4, NF-κB, MAPK	In vivo	[115]
		PF	ASIC3, ERK; Gas6, Axl, SOCS3, p-ASK1; TLR4, MyD88, TRIF;	In vivo	[118,119]
		PF	AChE, BuChE;	In vitro	[123]
Core component set for regulating qi and relieving pain	Disordered qi dynamics and endothelial dysfunction	NRG	AMPKα, Sirt1, eNOS; ATF4, CHOP;	In vivo	[125,139]
		NRG	STIM1, ORAI1; cGMP, VASP; miR-223-3p, IGF1R; PI3K, AKT, CREB5;	In vitro	[124,131,140,141]
		NAR	Sema3A; ERK, NF-κB; AT1R, PKC, NOX2, Raf-1, ERK1/2;	In vivo	[134,135]
		NAR	PDI;	In vitro	[132]
		HSD	CaMKII, p38 MAPK, eNOS; VEGFA, PI3K, AKT	In vitro	[138,143]
		FNT	ERK5, CD36;	In vivo	[133]
		PD	Ca^2+^, CaMKKβ, AMPK, CaMKIIα;	In vitro	[127]
		ISL	IL-10, SOD1, Nrf2, HO-1;	In vivo	[128]
		ISL	SIRT6, NLRP3;	In vitro	[129]
		ROF	MAPK-8, TRAF-6, TRAF-4, PDX-1, SIRT-1, INS-1, GLUT-4;	In vivo	[142]
	Inflammatory responses and immune dysregulation	NRG	TLR4, p47phox; TAK1, JNK, ERK, FoxO3a;	In vivo	[144,146]
		NAR	NF-κB, IL-17, caspase-3; PPARγ, miR-21;	In vivo	[159,162]
		NAR	p-STAT3, p-JAK, IL-10, IGF-1, arginase 1; MSR1, CD36, ABCA1, ABCG1, SR-B1; *miR-126*, GSK-3β;	In vitro	[147,160,161]
		NR	TXNIP, NLRP3;	In vivo	[154,155]
		FNT	α7nAChR, JAK2, STAT3; TLR4, NF-κB;	In vitro	[148,151]
		FNT	PTP1B, STAT6; GSK-3β;	In vivo	[149,150]
		NOB	TLR4, NF-κB, Keap1, Nrf2; IL-6, STAT3, FOXO3a;	In vitro	[152,153]
		PD	PI3K, AKT, NLRP3;	In vivo	[158]
		ROF	NF-Κb, HO-1, GSH, PPARα, SREBP1;	In vivo	[163]
		LIQ	NF-κB, p-p65; Collagen I, Collagen III, TGF-β1, MMP-9, α-SMA, CCL5, p-NF-κB; AMPKα2, mTORC1, IκBα, NFκB, p65;	In vivo	[164,165,166]
		LTG	TGF-β1, Smad2;	In vivo	[167]
		Isoquercitrin	HSP90, SGT1, NLRP3; *miR-138-5p*, PGC-1α;	In vivo	[156,157]
	Oxidative stress and mitochondrial dysfunction	NRG	SIRT1, FOXO3a, PGC-1α; GSH, LOOH, CAT, GST; CaMKII, Bcl-2, Drp1; Nrf2, HO-1, NLRP3; AMPK, Akt, mTOR, Keap1; YAP, STAT3;	In vivo	[168,169,176,177,178,194]
		NRG	SIRT1, BDNF; Bax, Bcl-2;	In vitro	[174,192]
		NAR	GSH, CAT, MDA; LKB1, AMPK, PGC-1α, Nrf2, NQO1; IRF3, SLC7A11, GPX4; DRP1, LRRK2, MCU;	In vivo	[170,175,191,201]
		NAR	HO-1, GPX4, NQO1, GSH, SOD; HIF-1α, BNIP3;	In vitro	[171,193]
		LIQ	TNFR1, NF-κB, MMP9; Keap1, Nrf2;	In vitro	[173,182]
		Isoquercitrin	Nrf2, HO-1, NLRP3, Caspase-1;	In vivo	[179]
		Isoquercitrin	LKB1, AMPK, ACC; Bax, Caspase-3, CytoC, Bcl-2;	In vitro	[196,200]
		NOB	Nrf2, SREBP-1c, NF-κB; Hippo, YAP;	In vivo	[180,195]
		HSD	Nrf2, NF-κB;	In vivo	[181]
		HSD	caspase-9, caspase-3	In vitro	[199]
		ISL	SIRT1, Nrf2; Keap1, ARE; HO-1, GPX4, SLC7A11; ANXA2, STAT3, NF-κB; PI3K, AKT, mTOR;	In vivo	[183,184,185,186,187]
		FNT	YAP;	In vivo	[197]
		PD	AMPK, PINK1, Parkin;	In vivo	[204]
	Qi-transforming function in energy and metabolism	NRG	CaMKKβ, AMPK, ACC; Pdx1, MafA; AMPK, Txnip; LKB1, PGC-1α, NF-κB, SIRT1;	In vivo	[209,210,211,212]
		NRG	GSH-Px, SOD, CAT, ROS, MDA, L-type Ca^2+^ currents; PPAR-α, CPT1; PPARγ, p38 MAPK;	In vitro	[205,213,244]
		NAR	TFEB; FXR, FGF15, CYP7A1, PCSK9, IDOL;	In vivo	[214,219]
		HSD	SIRT1, PGC-1α; AMPK; FASN, SCD1, HK2, ENO1, PI3K p110δ;	In vivo	[215,216,233]
		Isoquercitrin	GSTP, AMPK, SREBP-1c;	In vivo	[217]
		FNT	SIRT1, PGC-1α, PPARα;	In vivo	[218]
		ISL	NPC1L1;	In vitro	[220]
		LIQ	SIRT1, FXR, Nrf2	In vivo	[221]
		NOB	ANGPTL3, LXRα; CYP7A1; SIRT1, FGF21, ACC, FASN, CD36; Bmal1; AdipoR1, gp91^phox^;	In vivo	[224,225,226,229,230]
		NOB	PPARα; PPARG, CD36; *miR-433*, SIRT1; Cebpb, Pparg, Lpl, Scd1, Fas, IκBα;	In vitro	[222,223,227,228]
		LTG	mTOR, PI3K, Akt;	In vitro	[235,236]
		ISL	PYGL;	In vivo	[238]
		ISL	IQGAP2, CREB, SIRT1;	In vitro	[239]
		PD	AMPK, PCK1, G6Pase;	In vivo	[242]
		SSa	AMPK, ACC, ERK1/2, p38 MAPK;	In vitro	[243]
	Neural regulation and ion signaling	NRG	MAO-A, 5-HT, MAO-B; SYP, PSD95, NMDAR subunits;	In vivo	[245,247]
		NOB	GDNF, AKT, FOXO3a, P21;	In vivo	[246]
		ISL	GABA_B, P/Q-type calcium channels, PKC;	In vivo	[249]
		LIQ	Camp, PKA, LKB1, AMPKα2, mTORC1;	In vivo	[251]
		Isoquercitrin	NLRP3, Piezo1;	In vivo	[253]
	Gut microecology	NRG	ZO-1, occludin, claudin-5, TLR4, NF-κB, NLRP3; SCFA levels;	In vivo	[255,256]
		NOB	SCFA and BA metabolism; GM; BSH, FXR, SHP; ZO-1, Occludin;	In vivo	[258,260,262,263]
		HSD	GM	In vivo	[264]
		NAR	TLR4, p38 MAPK, NF-κB, Nrf2, cGAS, STING;	In vivo	[266]
		NR	claudin-3, occludin, ZO-1, TLR4, MAPK, NF-Κb;	In vivo	[267]
		Isoquercitrin	TLR4, MyD88, NF-κB;	In vivo	[268]
		ROF	GM, Th17, Treg	In vivo	[271]
		ISL	GM	In vivo	[272]
		FNT	GM, SCFA, Muc-2, occludin;	In vivo	[273,274]
		SSa	GM, FGF19, FXR, ASBT; 25-OHC, NLRP3	In vivo	[275,276]

## Data Availability

No new data were created or analyzed in this study.

## Abbreviations

Abbreviations	Name
ACEIs	Angiotensin-converting enzyme inhibitors
AGEs	Advanced glycation end products
ALB	Albiflorin
AMY	Amygdalin
Ang II	Angiotensin II
APD	Action potential duration
ARBs	Angiotensin receptor blockers
AREs	Antioxidant response elements
AS	Atherosclerosis
CHD	Coronary heart disease
CPBEB	Core set for the promotion of blood circulation and the elimination of blood stasis
CPT1	Carnitine palmitoyltransferase 1
CRQRP	Core set for the regulation of qi and the relief of pain
CVDs	Cardiovascular diseases
EndMT	Endothelial–mesenchymal transition
ER	Endoplasmic reticulum
ERβ	Estrogen receptor β
ET-1	Endothelin-1
FA	Ferulic acid
FASN	Fatty acid synthase
FFAs	Free fatty acids
FNT	Formononetin
FXR	Farnesoid X receptor
Gas6	Growth arrest-specific protein 6
GIP	Glucose-dependent insulinotropic polypeptide
GM	Gut microbiota
GRo	Ginsenoside Ro
GSDMD	Gasdermin D
HATs	Histone acetyltransferases
HCAECs	Human coronary artery endothelial cells
Hcy	Homocysteine
HDACs	Histone deacetylases
HFD	High-fat diet
HK1	Hexokinase 1
HSD	Hesperidin
HSYA	Hydroxysafflor yellow A
I/R	Ischemia–reperfusion
ICAM-1	Intercellular adhesion molecule-1
ISL	Isoliquiritigenin
LIQ	Liquiritin
lncRNA	Long noncoding RNA
LPS	Lipopolysaccharide
LTG	Liquiritigenin
MAFLD	Metabolic dysfunction-associated fatty liver disease
MDH1	Malate dehydrogenase 1
MHC II	Major histocompatibility complex class II
MI	Myocardial infarction
MMPs	Matrix metalloproteinases
MPO	Myeloperoxidase
MPT	Mitochondrial permeability transition
mPTP	Mitochondrial permeability transition pore
NAR	Naringin
NETs	Neutrophil extracellular traps
NHP	Neohesperidin
NOB	Nobiletin
NR	Narirutin
NRG	Naringenin
ox-LDL	Oxidized low-density lipoprotein
PAF	Platelet-activating factor
p-ASK1	Phosphorylated apoptosis signal-regulating kinase 1
PD	Platycodin D
PDGF	Platelet-derived growth factor
PDI	Protein disulfide isomerase
PF	Paeoniflorin
PKA	Protein kinase A
PKC	Protein kinase C
PLCγ2	Phospholipase Cγ2
PYGL	Glycogen phosphorylase L
RAAS	Renin–angiotensin–aldosterone system
ROF	Rhoifolin
ROS	Reactive oxygen species
SCFAs	Short-chain fatty acids
SOCE	Store-operated calcium entry
SphK1/2	Sphingosine kinases 1/2
SR	Sarcoplasmic reticulum
SR-A	Scavenger receptor-A
SSa	Saikosaponin A
TCM	Traditional Chinese medicine
Th1	T helper 1
TLRs	Toll-like receptors
TMA	Trimethylamine
TMAO	Trimethylamine N-oxide
VCAM-1	Vascular cell adhesion molecule-1
VEGF	Vascular endothelial growth factor
VLDL	Very-low-density lipoprotein
VSMCs	Vascular smooth muscle cells
vWF	von Willebrand factor
XFZYD	Xuefu Zhuyu Decoction
*β*-ECD	*β*-ecdysterone
